# Temporally degradable collagen–mimetic hydrogels tuned to chondrogenesis of human mesenchymal stem cells

**DOI:** 10.1016/j.biomaterials.2016.05.011

**Published:** 2016-08

**Authors:** Paresh A. Parmar, Stacey C. Skaalure, Lesley W. Chow, Jean-Philippe St-Pierre, Violet Stoichevska, Yong Y. Peng, Jerome A. Werkmeister, John A.M. Ramshaw, Molly M. Stevens

**Affiliations:** a Department of Materials, Imperial College London, Exhibition Road, London SW7 2AZ, United Kingdom; b Department of Bioengineering, Imperial College London, Exhibition Road, London SW7 2AZ, United Kingdom; c Institute of Biomedical Engineering, Imperial College London, Exhibition Road, London SW7 2AZ, United Kingdom; d CSIRO Manufacturing, Bayview Avenue, Clayton, Victoria 3169, Australia

**Keywords:** Hydrogel, Mesenchymal stem cell, Biodegradation, Biomimetic material, Bacterial collagen, Cartilage tissue engineering

## Abstract

Tissue engineering strategies for repairing and regenerating articular cartilage face critical challenges to recapitulate the dynamic and complex biochemical microenvironment of native tissues. One approach to mimic the biochemical complexity of articular cartilage is through the use of recombinant bacterial collagens as they provide a well–defined biological ‘blank template’ that can be modified to incorporate bioactive and biodegradable peptide sequences within a precisely defined three–dimensional system. We customized the backbone of a Streptococcal collagen–like 2 (Scl2) protein with heparin–binding, integrin–binding, and hyaluronic acid–binding peptide sequences previously shown to modulate chondrogenesis and then cross–linked the recombinant Scl2 protein with a combination of matrix metalloproteinase 7 (MMP7)– and aggrecanase (ADAMTS4)–cleavable peptides at varying ratios to form biodegradable hydrogels with degradation characteristics matching the temporal expression pattern of these enzymes in human mesenchymal stem cells (hMSCs) during chondrogenesis. hMSCs encapsulated within the hydrogels cross–linked with both degradable peptides exhibited enhanced chondrogenic characteristics as demonstrated by gene expression and extracellular matrix deposition compared to the hydrogels cross–linked with a single peptide. Additionally, these combined peptide hydrogels displayed increased MMP7 and ADAMTS4 activities and yet increased compression moduli after 6 weeks, suggesting a positive correlation between the degradation of the hydrogels and the accumulation of matrix by hMSCs undergoing chondrogenesis. Our results suggest that including dual degradation motifs designed to respond to enzymatic activity of hMSCs going through chondrogenic differentiation led to improvements in chondrogenesis. Our hydrogel system demonstrates a bimodal enzymatically degradable biological platform that can mimic native cellular processes in a temporal manner. As such, this novel collagen–mimetic protein, cross–linked via multiple enzymatically degradable peptides, provides a highly adaptable and well defined platform to recapitulate a high degree of biological complexity, which could be applicable to numerous tissue engineering and regenerative medicine applications.

## 1 Introduction

Developing bioengineered constructs for cartilage tissue engineering that promote neocartilage regeneration by encapsulated cells, while restoring both biomechanical and biochemical function in damaged tissues, remains a significant engineering challenge. Human mesenchymal stem cells (hMSCs) provide an autologous cell source that can be used to heal large osteoarthritic cartilage lesions [1] and can be induced to undergo chondrogenesis when subjected to specific biochemical and physical cues [2]. Three–dimensional (3D) environments have been shown to promote chondrogenesis of hMSCs [3], [4], [5], [6], [7]. Due to the importance of culturing hMSCs in a 3D environment, a large number of studies have been conducted with MSCs encapsulated in various types of hydrogel scaffolds based on both synthetic and naturally–derived materials including hyaluronic acid (HA) [3], poly(ethylene glycol) (PEG) [4], agarose [5], alginate [6], and collagen [7], with each presenting different advantages and disadvantages.

Hydrogels are particularly attractive cell carriers because their elasticity and highly aqueous environments permit extracellular matrix (ECM) evolution and nutrient transport, while physically entrapping cells into the cross–linked network [8], [9]. Additionally, biodegradation is ideal to allow for cellular migration and progressive replacement of the template material by neotissue deposition. Cell–mediated degradation occurs in hydrogels made of naturally derived materials such as collagen and fibrin, and these native molecules present moieties that can enhance cellular adhesion and biorecognition [6]. However, it is difficult to fabricate reproducible, mechanically robust, or tunable scaffolds using natural materials [8]. The ability to tune the degradation of the hydrogel with the deposition of ECM by harnessing cell–mediated processes has been shown to elicit advantages towards chondrogenesis. Hydrogels can be programmed to degrade by cell–mediated mechanisms, such as PEG hydrogels formed with matrix metalloproteinase (MMP)–degradable cross–links that were previously found to improve chondrogenesis of encapsulated hMSCs compared to non–enzymatically degradable PEG hydrogels [10]. Hydrogels can also provide a well–defined system that increases their feasibility for clinical translation while recapitulating features of natural systems such as cell–mediated degradation and adhesion to ECM molecules.

Recombinantly synthesized bacterial collagens, such as Streptococcus collagen–like 2 (Scl2), have recently been explored for numerous applications in tissue engineering and regenerative medicine [11], [12], [13], [14]. Scl2 proteins have been investigated largely due to their modularity and ability to be manipulated via chemical functionalization of amino acid residues or site–directed mutagenesis of selected peptide sequences to their backbone enabling the tuning of specific cellular processes [11], [14], [15]. Scl2 proteins contain the characteristic (Gly–Xaa–Yaa)_n_ repeating sequence that self–assembles into a triple helical conformation much like mammalian collagens [12], [13], [16]. In contrast to mammalian collagens, however, bioactive epitopes that interact with cells are not found on Scl2 proteins and thus, Scl2 proteins offer a well–defined biological ‘blank template’ onto which it is possible to selectively and systematically integrate specific bioactive motifs [11], [14], [15]. Another advantageous feature of Scl2 proteins is their non–immunogenicity and non–cytotoxicity as shown in a previous study as well as the ability to produce these proteins at high yields [12], [13], [15]. In addition, no post–translational modification of Scl2 proteins is required, resulting in minimal batch–to–batch variation in performance, quality, and purity. Taken together, recombinantly synthesizing collagen–mimetic proteins may also reduce the overall production costs compared to naturally occurring mammalian collagens. Scl2 proteins have previously been used to generate Scl2–based hydrogels functionalized with glycosaminoglycan (GAG)–binding peptides and MMP7–cleavable peptide cross–links to promote the chondrogenic differentiation of encapsulated hMSCs [11]. Additionally, Scl2 proteins containing integrin–binding sequences have been combined with PEG to form hydrogels that effectively bind smooth muscle and endothelial cells for engineering vascular grafts [14].

The purpose of the current study was to build on the highly promising previous findings on Scl2–based hydrogels [11], which showed that recombinant Scl2 proteins containing HA–binding motifs enhanced chondrogenesis of encapsulated hMSCs to a greater extent than either RGDS peptides or chondroitin sulfate–binding motifs. Here, the backbone of Scl2 proteins was modified with HA–binding, heparin (H)–binding, and integrin (I)–binding sequences via site–directed mutagenesis to enhance biological recognition and adhesion of encapsulated hMSCs. The I-binding peptide sequence was included in the Scl2 protein constructs to aid initial cell adhesion to the scaffolds and maintain viability. The well–known RGD motif was not used in the present study since it has been shown to lead to hMSC hypertrophy in long–term culture [4]. As such, the I-binding peptide used in the present study was an alternative that does not include the RGD sequence. The inclusion of the HA–binding motif was verified in our previous work [11], and the reason for including all three of these biological motifs was to provide cell–adhesion and local presentation of biomolecules in order to create a hydrogel environment that mimics some of the characteristics of the highly complex cartilage tissue environment. It was hypothesized that chondrogenesis and cartilaginous matrix production would be further improved by implementing a combination of degradable peptides, where one peptide is targeted to an enzyme expressed by hMSCs undergoing chondrogenesis and the other susceptible to enzymes produced by the newly differentiated chondrocytes in order to trigger ongoing scaffold degradation. Thus, an MMP7–cleavable cross–linker to target enzymes produced during the early stages of chondrogenesis was combined with a peptide cross–linker that degraded specifically through the action of mature chondrocytes. Two aggrecanases (A Disintegrin And Metalloproteinase with ThromboSpondin motif (ADAMTS); ADAMTS4 and 5) were selected due to their increased activity relative to MMPs in mature cartilage tissue *in vivo* [17], [18] and inactivity during chondrogenesis before cells have committed to the chondrocyte lineage [19]. These two aggrecanases specifically cleave aggrecan (ACAN) at the TEGE–ARG site in the interglobular domain [20], and a previous study identified the ability of ADAMTS4 secreted by chondrocytes to cleave a novel peptide cross–linker containing that specific sequence. In that study, chondrocytes were encapsulated in photopolymerized 8–arm PEG hydrogels cross–linked with the ADAMTS4–cleavable peptide, and the chondrocyte phenotype (indicated by increased collagen type II deposition and decreased collagen type I and X) was improved with the novel ADAMTS4–cleavable cross–linker compared to non–degradable hydrogels [21]. Based on these observations, it was hypothesized that the ADAMTS4–cleavable cross–linker would improve chondrogenesis and cartilaginous matrix deposition by the encapsulated hMSCs. In this work, bioactive Scl2 proteins were cross–linked with peptides specific to MMP7, ADAMTS4, or various ratios of the two degradable cross–linkers, and the gene expression and matrix production of encapsulated hMSCs were observed throughout a 6 week culture period.

## 2 Materials and methods

### 2.1 Materials

Rink amide resin, Fmoc–protected amino acids, *N*, *N* dimethyl formamide (DMF), dichloromethane (DCM), 20% (v/v) piperidine in DMF, O–benzotriazole–*N,N,N*′,*N*′–tetramethyluronium–hexafluoro–phosphate (HBTU), and diisopropylethylamine (DIEA) were purchased from AGTC Bioproducts (UK). MMP7 and ADAMTS4 fluorogenic substrates were purchased from Merck Millipore (UK). All primary and secondary antibodies used for immunohistochemistry were purchased from Abcam (UK). All other chemicals were purchased from Sigma–Aldrich (UK). All chemicals were used as provided by the manufacturers. Recombinant Scl2 proteins were expressed in *Escherichia coli* BL21–DE3 and purified as described in Section 2.3.

### 2.2 Peptide synthesis and purification

The MMP7 (PLELRA)–, scrambled MMP7 (ScrMMP7; PALLRE)–, and ACAN (RDTEGEARGSVIDR)–cleavable peptides were synthesized manually on a 2 mmol scale using standard Fmoc solid phase peptide synthesis techniques as previously described [22]. For each coupling, the Fmoc protecting group was removed with 20% (v/v) piperidine in DMF followed by washing with DCM and DMF. Amino acids were activated by adding 4 molar equivalent of each Fmoc protected amino acid to 3.95 molar equivalent of HBTU and dissolved in DMF. Six molar equivalent of DIEA was added to the amino acid solution and the coupling solution added to the resin. The coupling reaction was allowed to proceed for two to three hours before the resin was washed in DCM and DMF. Ninhydrin tests were performed after each Fmoc deprotection and coupling step to monitor the presence of free amines.

Fmoc–Lys(Mtt)–OH was also coupled to the peptides at the N and C termini using methods described above. The ninhydrin test for free amines was conducted at room temperature for 30 min since the Mtt protecting group is heat–labile. The Mtt protecting group was removed from the amine on the lysine side chain with 5% (v/v) TFA in DCM for 2 min on the shaker. The solution turns bright yellow as the Mtt is removed, and this was repeated at least 5 times or until the solution was clear. The beads were washed thoroughly with DCM and DMF before running the ninhydrin test at room temperature for 30 min. To attach acrylate groups to the peptide, acryloyl chloride was activated by adding 10 molar equivalents of acryloyl chloride and 10 molar equivalents of DIEA in DCM. The coupling was verified with the ninhydrin test at room temperature for 30 min.

Once the synthesis was completed, the peptides were cleaved in 95% (v/v) trifluoroacetic acid (TFA), 2.5% (v/v) triisopropyl silane (TIS), and 2.5% (v/v) H_2_O for four hours. TFA was removed using rotary evaporation, and the peptide residues were precipitated and washed with cold diethyl ether by centrifugation. The peptide precipitates were then allowed to dry under vacuum to remove residual ether. The peptides were purified (Fig. S1) using reverse phase preparative high performance liquid chromatography (HPLC; Shimadzu) in an acetonitrile/water gradient under acidic conditions on a Phenomenex C18 Gemini NX column (5 μm pore size, 110 Å particle size, 150 × 21.2 mm). Following purification, the peptides were lyophilized on a freeze dryer (Labconco) for storage at 4 °C prior to use. The purified peptide masses were verified by matrix assisted laser desorption spectroscopy (MALDI; Waters).

### 2.3 Streptoccocal collagen–like 2 protein synthesis and purification

**Fig. 1:**
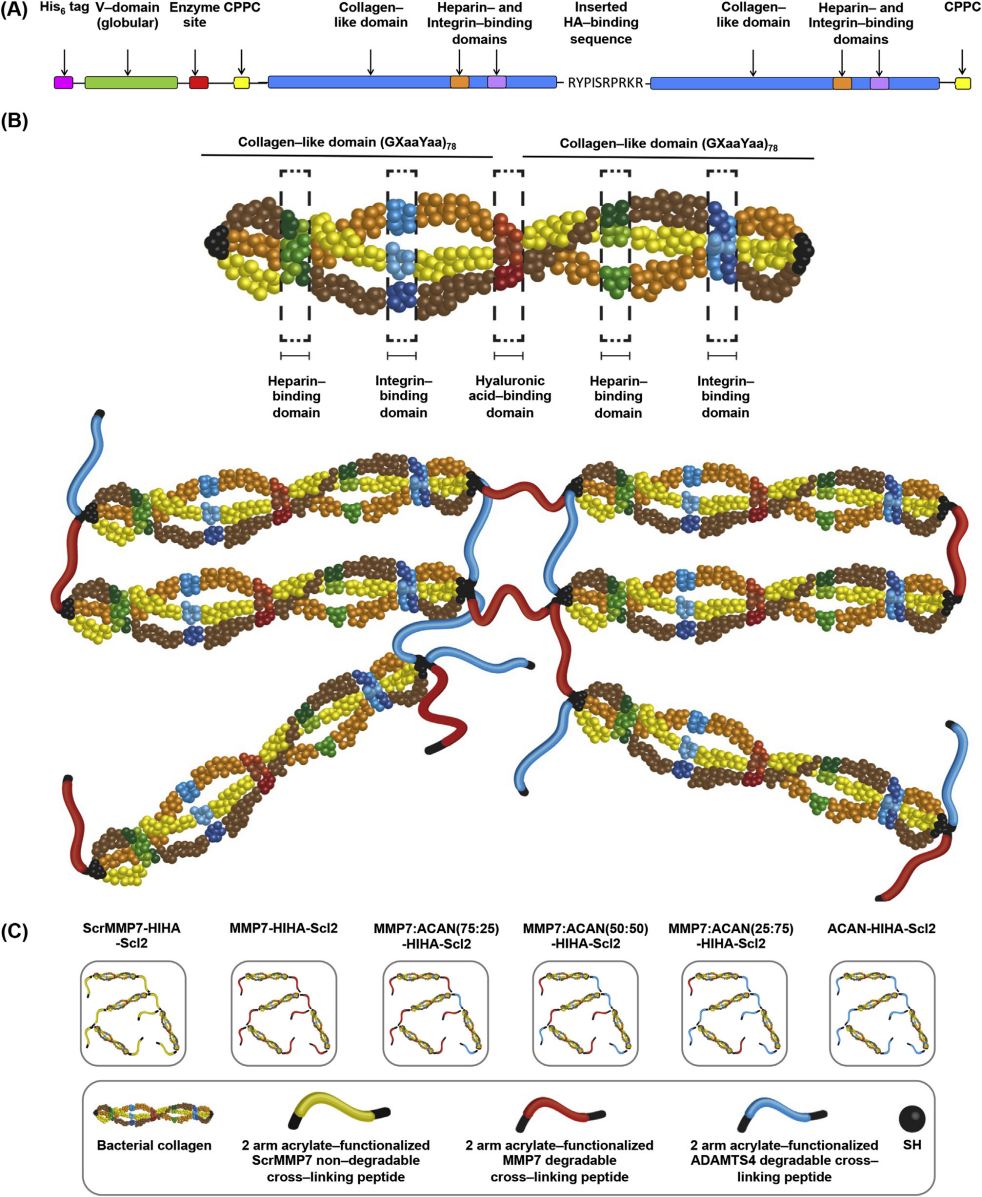
Schematic diagrams of the peptide–functionalized Scl2 protein and hydrogel. (A) Schematic diagram of the Scl2 protein containing H–binding, I–binding, and HA–binding peptide sequences. The ‘CPPC’ domains represent the amino acid sequences inserted at the N and C termini to aid stability of each construct. (B) Generalized structure of the peptide–functionalized Scl2 protein and hydrogel. For clarity, the homotrimer chains are represented as different colors, and the triple helical motif has been opened out to show the three chains. The locations of the H–binding and I–binding sites are not proportional to their locations in the construct. The 2–arm acrylate–functionalized MMP7 and ACAN degradable peptides were conjugated to the Scl2 protein via thiol–acrylate reactions to generate hydrogels. (C) Schematic diagrams of different Scl2 protein hydrogel samples.

The gene constructs used were based on the DNA sequence for the fragment of the *Scl2.28* allele (Q8RLX7) of *Streptococcus pyogenes* encoding the combined globular and collagen–like portions of the *Scl2.28* protein, but lacking the C terminal attachment domain as previously described [12], [13], [15], [23]. Constructs included an additional enzyme cleavage and spacer sequence LVPRGSP between the N terminal globular domain (V) and the following (Gly–Xaa–Yaa)_n_ collagen–like (CL) domain sequences. The new construct for the present study, HIHA-Scl2, contained a HA–binding (RYPISRPRKR) sequence between two CL domains (Fig. 1). In addition, each of the two CL domains included H–binding (GRPGKRGKQGQK) and I–binding (GERGFPGERGVE) sequences integrated within the triple helical structure, as previously described [15]. The C terminal of this initial construct had an additional C terminal GGPCPPC sequence. This DNA sequence was synthesized commercially with codon optimization for expression in *E. Coli* (GeneArt^®^ Gene Synthesis, Germany). Subsequently, in order to further stabilize the triple helix and allow for subsequent functionalization via thiol–acrylate chemistry, an additional GGPCPPC sequence was added at the N terminal between the spacer sequence and the CL domain, using the QuikChange site–directed mutagenesis kit (Stratagene) according to the manufacturers' instructions and synthesized primers (Integrated DNA Technologies). The sequences of the initial and final constructs were confirmed by sequencing prior to transformation and protein expression.

The final DNA sequences were sub–cloned into the pColdI (Takara Bio, Japan) vector systems for expression in *E. coli* (Fig. S2A). The constructs did not include an N terminal His_6_–tag, which was provided by the pColdI vector [12], [13], [15], [23]. For protein production, a selected positive clone was transformed and then expanded in flask culture. The pColdI constructs were expressed in the *E. coli* BL21–DE3 strain. Cells were grown in 2× yeast extract–tryptone (YT) media with ampicillin (100 μg/mL) at 37 °C, with shaking at 200 rpm until the A600 absorbance reading reached an optical density in the range 3–6 A.U. Cells were then cooled to 25 °C and 1 mM isopropyl β–d–thiogalactopyranoside was added to induce protein expression. After 10 h incubation, cells were further cooled to 15 °C for 14 h, after which the cells were harvested by centrifugation (12,000 g, 60 min) at 4 °C. For protein extraction, each 1 g of cell paste was re–suspended in 20 mL of 20 mM sodium phosphate buffer at pH 8.0 and the cells ruptured by sonication on ice using a Misonix S4000 instrument with a Enhance Booster #1 probe [12], [13], [15], [23]. Clarified lysate (12,000 g for 30 min, 4 °C) was adjusted to pH 2.2 and held at 4 °C for 16 h. Any precipitate that had formed was removed (12,000 g for 30 min, 4 °C) and the supernatant, containing the expressed collagens, was treated by pepsin (0.01 mg/mL) for 16 h at 4 °C [23]. Collagens were concentrated and buffer exchanged into 20 mM sodium phosphate buffer, pH 8.0 using a 10 kDa cross–flow filtration membrane (Pall Life Sciences). Purity was verified by 12% sodium dodecyl sulfate–polyacrylamide gel electrophoresis (SDS–PAGE) (Fig. S2B) and MALDI [12], [13], [15].

### 2.4 Characterization of functionalized Scl2 proteins

HIHA-Scl2 was characterized using Fourier transform infrared (FTIR) spectroscopy to evaluate the conjugation chemistry. FTIR analysis with a Perkin Elmer Spectrum One spectrometer was used to evaluate the conjugation of the cross–linking peptides to Scl2 protein as previously described [14]. FTIR spectra were taken with a scanning wavenumber range from 4000 to 650 cm^−1^.

### 2.5 Preparation of Scl2 hydrogels

To generate hydrogels, HIHA-Scl2 protein was re–suspended at 100 mg/mL in chondrogenic medium (medium defined in Section 2.8) at room temperature. The MMP7–cleavable, non–cleavable ScrMMP7, or ACAN–cleavable peptides were dissolved in 4 mM triethanolamine (TEA) in chondrogenic medium and equimolar equivalents of these peptides were reacted to modify 100% of the thiols on the HIHA-Scl2 protein. The MMP7– and ACAN–cleavable peptides were mixed in a 75:25, 50:50, and 25:75 M ratio. The resulting solutions were sterile–filtered and pipetted to generate homogeneous formation of ScrMMP7-HIHA-Scl2, MMP7-HIHA-Scl2, MMP7:ACAN(75:25)-HIHA-Scl2, MMP7:ACAN(50:50)-HIHA-Scl2, MMP7:ACAN(25:75)-HIHA-Scl2, and ACAN-HIHA-Scl2 hydrogels.

### 2.6 Hydrogel characterization

#### 2.6.1 Morphological characterization

Hydrogels were imaged by multi–photon second harmonic generation (MP–SHG) in wet state in PBS using a Leica SP5 inverted microscope equipped with a MaiTai HP DeepSee multi–photon laser (Spectraphysics) on a 25× NA objective. Second harmonic signal was generated at 900 nm and detected on a photomultiplier tube (PMT) (435–465 nm).

#### 2.6.2 Mechanical characterization

Mechanical properties of the hydrogels were studied using oscillatory shear rheology. Oscillatory parallel plate rheological measurements were performed using an Advanced Rheometer AR2000ex with AR Instrument Software (TA instruments) fitted with a Peltier temperature control system. Samples were tested at 37 °C using an 8 mm diameter parallel steel plate. All samples were individually prepared immediately prior to testing. Two sequential sweeps were applied; (1) time sweep for 2 h at 0.1% strain and 6.28 rad/s angular frequency and (2) strain sweep from 0.01 to 100% at 6.28 rad/s angular frequency. For all samples, a compression load of 0.5 N was exerted during testing. Hydrogels were also studied using dynamic mechanical analysis (DMA) in unconfined compression mode. For all tests, samples were incubated in PBS for 24 h prior to testing to ensure samples were in an equilibrium state and dimensions were measured in wet state using digital calipers. Hydrogels were mechanically tested in compression using a Bose Electroforce testing machine equipped with a 22.5 N load cell. Samples were incubated in PBS, pre–loaded to 0.05 N, compressed to 10% strain at a cross–head speed of 0.5% strain/min, and then followed by a frequency sweep from 0.1 Hz to 10 Hz. The compressive modulus was calculated from the linear portion of the stress–strain curve.

#### 2.6.3 GAG binding

The effectiveness of the H–binding and HA–binding peptide sequences incorporated in the Scl2 backbone at binding and retaining specific GAGs was evaluated using fluorescein isothiocyanate (FITC)–labeled H and HA (Creative PEGWorks, UK). Scl2 proteins were coated onto 96–well plates, incubated at 37 °C for 24 h, washed three times in PBS, incubated in 1% (w/v) bovine serum albumin (BSA) in PBS for 5 h, washed three times in PBS, incubated in 0.5 mg/mL FITC–labeled H or HA for 24 h, washed three times in PBS to remove unbound fluorescent GAGs, and stored in PBS at 37 °C between measurements. To study the release of H or HA from the hydrogels, they were processed exactly as protein–coated wells. After 1, 3, and 7 days, PBS was removed and fluorescence intensities of the supernatants were measured to evaluate GAG binding and retention. Samples were excited at 552 nm or 485 nm, and the fluorescence emission intensity was measured at 575 nm or 525 nm for FITC–labeled H or HA, respectively. Samples were incubated in fresh PBS at 37 °C between readings. The fluorescence was measured in arbitrary units and relative binding of H or HA was normalized to the highest level of fluorescent intensity at each time point.

#### 2.6.4 Swelling behavior

The swelling behavior of hydrogels was measured by recording their dry weight (W_d_) followed by swelling in 0.5 mg/mL H or HA in PBS for 24 h at 37 °C. The hydrogels were then removed from solution at 2, 4, 6, 24, and 48 h post incubation, washed three times in PBS, and their wet weight (W_s_) was recorded. The swelling ratio of the hydrogels was calculated using Equation (1) and normalized to their swelling ratio in PBS without exogenous GAGs in the solution.

(1) $$\text{Swelling}\;\text{ratio}\left(\text{\%}\right)=\left[\frac{\left(\text{Ws}-\text{Wd}\right)}{\text{Wd}}\right]\times 100$$

### 2.7 hMSC culture

Bone marrow–derived hMSCs were purchased from PromoCell GmbH (Germany). The multilineage differentiation capacity of hMSCs was verified by PromoCell GmbH prior to purchase. hMSCs were seeded at 4000 cells per cm^2^ in T225 flasks and cultured in mesenchymal stem cell growth medium (MSCGM) (PromoCell GmbH, Germany). hMSCs were incubated at 37 °C in a 5% CO_2_ atmosphere and the medium was changed every three days. The cells were harvested at 80% confluency with 0.025% (w/v) trypsin–EDTA in PBS, centrifuged, and sub–cultured in MSCGM. Passage 6 hMSCs were used for all cell response experiments.

### 2.8 Cell seeding and culture

hMSCs were homogeneously dispersed at 8 × 10^6^ cells per mL in pre–made 100 mg/mL HIHA-Scl2 solution containing chondrogenic medium (high–glucose (4.5 g/L) Dulbecco's Modified Eagle Medium (DMEM; Invitrogen, UK) supplemented with 0.1 mM dexamethasone, 1% (v/v) penicillin streptomycin, 50 μg/mL l–proline, 50 μg/mL ascorbate–2–phosphate, 1× insulin–transferrin–selenium (ITS) Premix (BD Biosciences, UK), and 10 ng/mL TGF–β3 (Lonza, UK)). The MMP7, ScrMMP7, or ACAN peptides were prepared in 4 mM TEA in chondrogenic medium and mixed with the protein/cell solution. Aliquots (50 μL) of the resulting mixture were pipetted in a non–tissue culture treated 48–well plate and allowed to gel for 30 min at 37 °C in a 5% CO_2_ atmosphere before slowly topping up the wells with 1 mL of chondrogenic medium. Hydrogels were incubated at 37 °C in a 5% CO_2_ atmosphere for six weeks with the medium changed every three days.

Additionally, hMSCs were used in a pellet culture system. hMSCs were homogeneously dispersed at 2.5 × 10^5^ cells per mL in 1 mL chondrogenic medium and centrifuged (400 g, 10 min) at 37 °C in a 15 mL polypropylene conical tube. Pelleted cells were cultured for two weeks at 37 °C in a 5% CO_2_ atmosphere with loosened caps to allow gas exchange and with the medium changed every three days.

### 2.9 Weight change and enzymatic activity assays

The dry weight of each hydrogel was measured over time in absence of cells to evaluate their degradation *in vitro*. Hydrogels were prepared as previously described and incubated in chondrogenic medium for 24 h at 37 °C in a 5% CO_2_ atmosphere. The hydrogels were then incubated in chondrogenic medium with exogenous enzymes (30 ng/mL) at 37 °C in a 5% CO_2_ atmosphere for one week with medium changed and dry weight measurements after lyophilization taken daily. Percentage weight change was normalized to day 0. Degradation by recombinant human MMP1, MMP2, MMP7, MMP13, and ADAMTS4 (AnaSpec, USA) was tested against a negative control (chondrogenic medium alone) and a positive control (0.2 μg/mL trypsin).

Cell–seeded hydrogels were incubated in chondrogenic medium at 37 °C in a 5% CO_2_ atmosphere for six weeks, and dry weight measurements after lyophilization were taken after 0, 1, 3, 7, 14, 21, 28, and 42 days of culture. Percentage weight change corresponding to the cumulative effect of cell proliferation, cartilage–like matrix deposition, and hydrogel degradation was normalized to day 0.

At each time point, 1 mL of medium was also removed, sterile–filtered, and analyzed for MMP7 and ADAMTS4 activities. MMP7 and ADAMTS4 activities were determined using fluorogenic MMP7 and ADAMTS4 substrate assays and compared to a negative control (chondrogenic medium) and a positive control (recombinant human MMP7 and ADAMTS4) according to the manufacturers' instructions.

### 2.10 Cell adhesion and viability

hMSC–seeded hydrogels were cultured for 0, 1, 3, 7, 14, 21, 28, and 42 days. After the culture period, the hydrogels were washed three times in PBS and analyzed for cell viability and adhesion. Cell viability was qualitatively assessed with a LIVE/DEAD^®^ Viability/Cytotoxicity Kit (Molecular Probes, USA) according to the manufacturers' instructions. Fluorescence confocal microscopy (Leica SP5 inverted microscope, Leica Microsystems) was used to visualize live (calcein; green) and dead (ethidium homodimer–1; red) cells. The metabolic activity of cells in the hydrogels was quantified by the AlamarBlue^®^ assay (Serotec, USA). This assay is based on the fluorescent signal output produced by metabolically active cells. Measurements were made at 570 nm and 600 nm. Cell–free hydrogels and empty wells were used as controls. All data were normalized to DNA content present (Section 2.11.) at each time point.

The effectiveness of the integrin–binding peptide sequence incorporated into the Scl2 backbone at binding hMSCs was evaluated using Scl2 proteins with (I-Scl2) and without (blank Scl2) the integrin–binding motif. Scl2 proteins were coated onto 96–well plates, incubated at 4 °C for 24 h, washed three times in PBS, incubated in 2% (w/v) BSA in PBS for 2 h, washed three times in PBS, and seeded with 2.5 × 10^5^ cells/cm^2^ in serum–free medium. The cells were incubated at 37 °C in a 5% CO_2_ atmosphere for 90 min and washed three times in PBS. 100 μL of MTS solution (2 mL 3–(4,5–dimethylthiazol–2–yl)–5(3–carboxymethoxphenyl)–2–(4–sulfophenyl)–2H–tetrazolium salt, 0.1 mL phenazine methosulfate, and 10 mL serum–free medium) (Abcam, UK) was added per well and samples were incubated at 37 °C in a 5% CO_2_ atmosphere for 90 min. Absorbance of the samples was read at 490 nm and normalized to binding of cells to rat tail collagen type I.

### 2.11 DNA, sGAG, and hydroxyproline quantification

hMSC–seeded hydrogels were cultured for 0, 1, 3, 7, 14, 21, 28, and 42 days. After the culture period, the constructs were washed three times in PBS and digested individually in papain digest solution (2.5 units papain/mL, 5 mM cysteine HCl, 5 mM EDTA, pH 7.4, in PBS) at 60 °C for 24 h. Papain digests were stored at −20 °C until further analysis. Digested samples were assayed for DNA content using the Quant–iT™ PicoGreen^®^ Kit (Invitrogen, UK) according to the manufacturers' instructions. Measurements were made at 535 nm. The standard curve was generated with dsDNA (Invitrogen, UK).

Sulfated GAG (sGAG) content was quantified using the Blyscan Kit (Biocolor, UK) according to the manufacturers' instructions. Measurements were made at 656 nm. The standard curve was generated with bovine trachea chondroitin sulfate A.

Total new, mammalian cell–derived collagen content was estimated by measuring the hydroxyproline content. Unlike mammalian collagens, bacterial collagens lack hydroxyproline, which enabled us to distinguish between the collagen in the hydrogel and new collagen deposition by the hMSCs. Papain–digested samples were hydrolyzed in 6 N HCl at 110 °C for 18 h. The hydroxyproline content of the hydrolysate was determined using the chloramine–T/Ehrlich's reagent assay and the color change quantified spectrophotometrically at 560 nm [24], [25]. The standard curve was generated with l–hydroxyproline and a conversion factor of 10 was used to convert from hydroxyproline to total collagen content.

### 2.12 Histology and immunohistochemistry

After 0 and 42 days of culture, hMSC–seeded hydrogels were washed three times in PBS, fixed with 4% (v/v) paraformaldehyde (Electron Microscopy Sciences, USA) for 30 min at 4 °C, washed three times in PBS, permeabilized with 0.4% (v/v) Triton X–100 for 30 min, and washed again. Hydrogels were flash frozen in OCT (Tissue–Tek, Fisher Scientific, UK) and cryosectioned at a thickness of 10 μm. Sections were transferred to treated slides (Superfrost Plus, Thermo Scientific, UK) and allowed to adhere for 24 h at 4 °C. Slides were stained for deposited sGAG with Alcian Blue (AB; pH 2.5) and for cell nuclei and matrix with Haematoxylin and Eosin (H&E).

Immunohistochemical staining (IHC) was performed for collagen type I, collagen type II, collagen type X, SOX9, Runx2, and PPAR-γ with rabbit IgG secondary antibody only and PBS negative controls. Samples were pre–treated with hydrogen peroxide, an avidin and biotin blocking kit (Vector Labs, UK), and blocked with 5% (v/v) goat serum. Primary antibodies were incubated overnight at 1/200 in 5% (v/v) goat serum, followed by goat anti–rabbit secondary antibody labeled with HRP at 1:100 for 1 h, stained with a 3,3′–diaminobenzidine (DAB) kit (Vector Labs, UK) for 10 min, and counter–stained with Haematoxylin. All stained sections were dehydrated, mounted with Histomount (Fisher Scientific, UK), and viewed on an Olympus BX51 microscope equipped with an Olympus DP70 camera. Sections stained with the SOX9 antibody were further examined to determine the extent of hMSC chondrogenesis by obtaining the number of nuclei stained positive for SOX9 at week 2 and normalizing to the total number of nuclei.

### 2.13 Gene expression analysis

hMSC–seeded hydrogels were cultured for 0, 1, 3, 7, 14, 21, 28, and 42 days. After the culture period, the constructs were washed three times in PBS. Total RNA was isolated using a tissue ruptor (Qiagen, USA) to homogenize samples with RLT buffer after which QIAshredder columns (Qiagen, USA) and the RNeasy Mini Kit (Qiagen, USA) were used to extract the RNA according to the manufacturers' instructions. QuantiTect^®^ Reverse Transcription Kit (Qiagen, USA) and QuantiTect^®^ SYBR Green PCR Kit (Qiagen, USA) were used to perform reverse transcription and quantitative PCR (qPCR), respectively. Thermocycling and SYBR Green detection were performed on a Corbett Rotorgene 6000 (Qiagen, USA) with extension at 72 °C and denaturing at 95 °C. Annealing temperatures were primer specific. Data were analyzed using the ΔΔCt method [26]. The following primers were used: MMP7 (Forward 5′–GAGTGAGCTACAGTGGGAACA–3′ and Reverse 5′–CTATGACGCGGGAGTTTAACAT–3′), ADAMTS4 (Forward 5′–GCAACGTCAAGGCTCCTCTT–3′ and Reverse 5′–CTCCACAAATCTACTCAGTGAAGCA–3′), TIMP2 (Forward 5′–TGGACGTTGGAGGAAAGAAG–3′ and Reverse 5′–GGGCACAATGAAGTCACAGA–3′), and TIMP3 (Forward 5′–CTGCTGACAGGTCGCGTC–3′ and Reverse 5′–CAACCCAGGTGATACCGATAGT–3′) at an annealing temperature of 52 °C, GAPDH (Quiagen, USA) (Forward 5′–TGGTATCGTGGAAGGACTCATGA–3′ and Reverse 5′–ATGCCAGTGAGCTTCCCGTTCAG–3′), COL1A1 (Forward 5′–CATTAGGGGTCACAATGGTC–3′ and Reverse 5′–TGGAGTTCCATTTTCACCAG–3′), COL2A1 (Forward 5′–CATCCCACCCTCTCACAGTT–3′ and Reverse 5′–GTCTCTGCCTTGACCCAAAG–3′), COL10A1 (Forward 5′–AATGCCTGTGTCTGCTTTTAC–3′ and Reverse 5′–ACAAGTAAAGATTCCAGTCCT–3′), and ACAN (Forward 5′–CACTGTTACCGCCACTTCCC–3′ and Reverse 5′–GACATCGTTCCACTCGCCCT–3′) at an annealing temperature of 60 °C, Runx2 (Forward 5′–CCGCCTCAGTGATTTAGGGC–3′ and Reverse 5′–GGGTCTGTAATCTGACTCTGTCC–3′) at an annealing temperature of 61 °C, and SOX9 (Forward 5′–AACGCCGAGCTCAGCAAG–3′ and Reverse 5′–ACGAACGGCCGCTTCTC–3′) at an annealing temperature of 62 °C.

### 2.14 Statistical analysis

All cell–related experiments were repeated three times with hMSCs from different donors and with an intra–experimental sample size of 3. Data are presented as means ± standard deviation (SD). Statistical significance was determined by performing analysis of variance (ANOVA) with Bonferroni correction and with a significance accepted at *p*–value < 0.05.

## 3 Results and discussion

### 3.1 Characterization of Scl2 proteins and acellular hydrogels

#### 3.1.1 Scl2 protein characterization

FTIR spectroscopy was used to confirm the conjugation of cross–linking peptides to Scl2 proteins (Fig. S3). It showed IR transmittance peaks at 1630 cm^−1^ (amide C=O) that were assigned to the Scl2 protein in all samples and used for normalizing. The peak at 1110 cm^−1^ (ether C—O—C) assigned to the acrylate was present in all samples except the HIHA-Scl2 control.

#### 3.1.2 Mechanical and morphological characterization of hydrogels

**Fig. 2:**
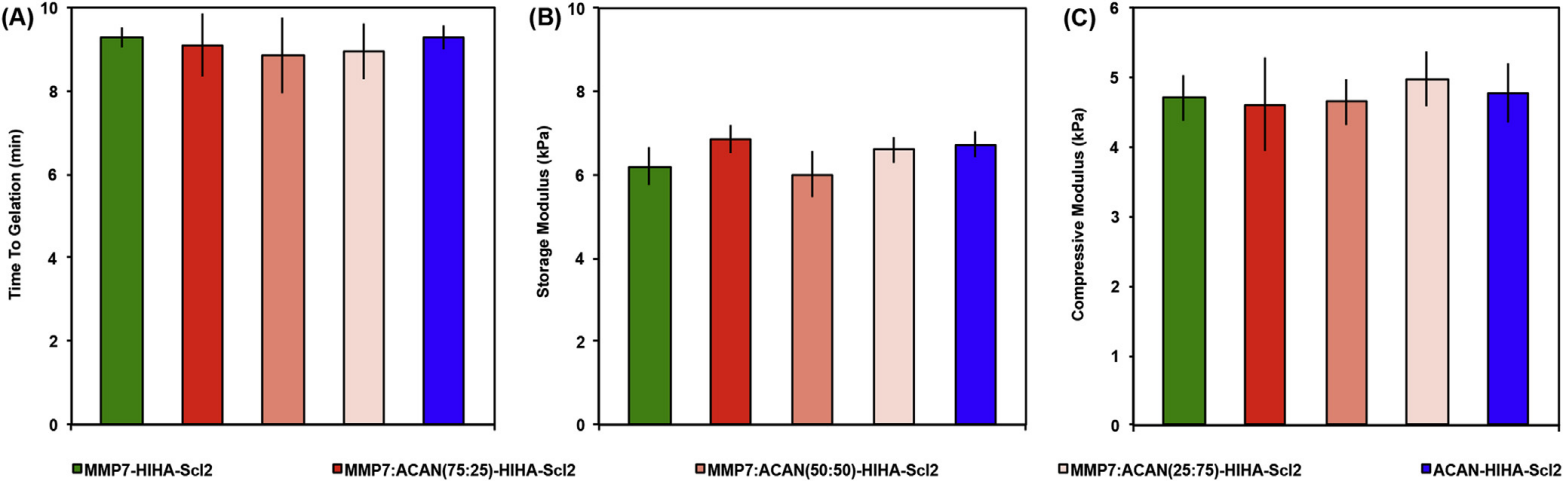
Mechanical properties of Scl2 hydrogels. (A) Time to gelation at a temperature of 37 °C, angular frequency of 6.28 rad/s, and strain of 0.5%. (B) Storage modulus determined from strain sweep up to 1% strain at a temperature of 37 °C and an angular frequency of 6.28 rad/s. (C) Dynamic mechanical analysis (DMA) used to determine the elastic modulus of compression of hydrogels compressed to 10% strain at 0.5% strain/min and 1 Hz. Values represent means ± SD (n = 3).

For all hydrogels, the ratio of the peptide cross–linkers was modified, however, the total degree of cross–linking was kept constant to maintain comparable mechanical properties and minimize any potential effects of matrix stiffness on cell behavior [27], [28]. Oscillatory shear rheology confirmed hydrogel formation and was used to measure the storage modulus (Fig. 2 and Fig. S4). No statistical differences in time to gelation and storage moduli were observed. The equilibrium storage moduli of all samples remained at ∼7 kPa in the linear elastic region up to 10% strain. Confirming these observations, the compression moduli of the hydrogels determined using unconfined DMA were found to range between ∼4 and 5 kPa at 1 Hz, and there were no statistical differences in compression moduli between samples. Unlike PEG, a commonly used hydrogel platform in cartilage tissue engineering, the Scl2 hydrogels exhibited viscoelastic behavior, which may be a more suitable replacement for tissues such as cartilage that also behave as viscoelastic materials [29]. The viscoelasticity is demonstrated by the fact that all hydrogels displayed increased compression moduli with increasing frequency from 0.1 Hz (∼3 kPa) to 10 Hz (∼6 kPa). Moreover, hydrogels imaged using MP–SHG showed no differences in morphology (Fig. S5). Together these observations indicate that the different cross–linking formulations did not affect the mechanical or structural properties of the hydrogels, and therefore we could compare the effects of the different degradable cross–linkers decoupled from the initial mechanical properties and hydrogel structure.

#### 3.1.3 GAG–binding properties of modified Scl2 proteins

**Fig. 3:**
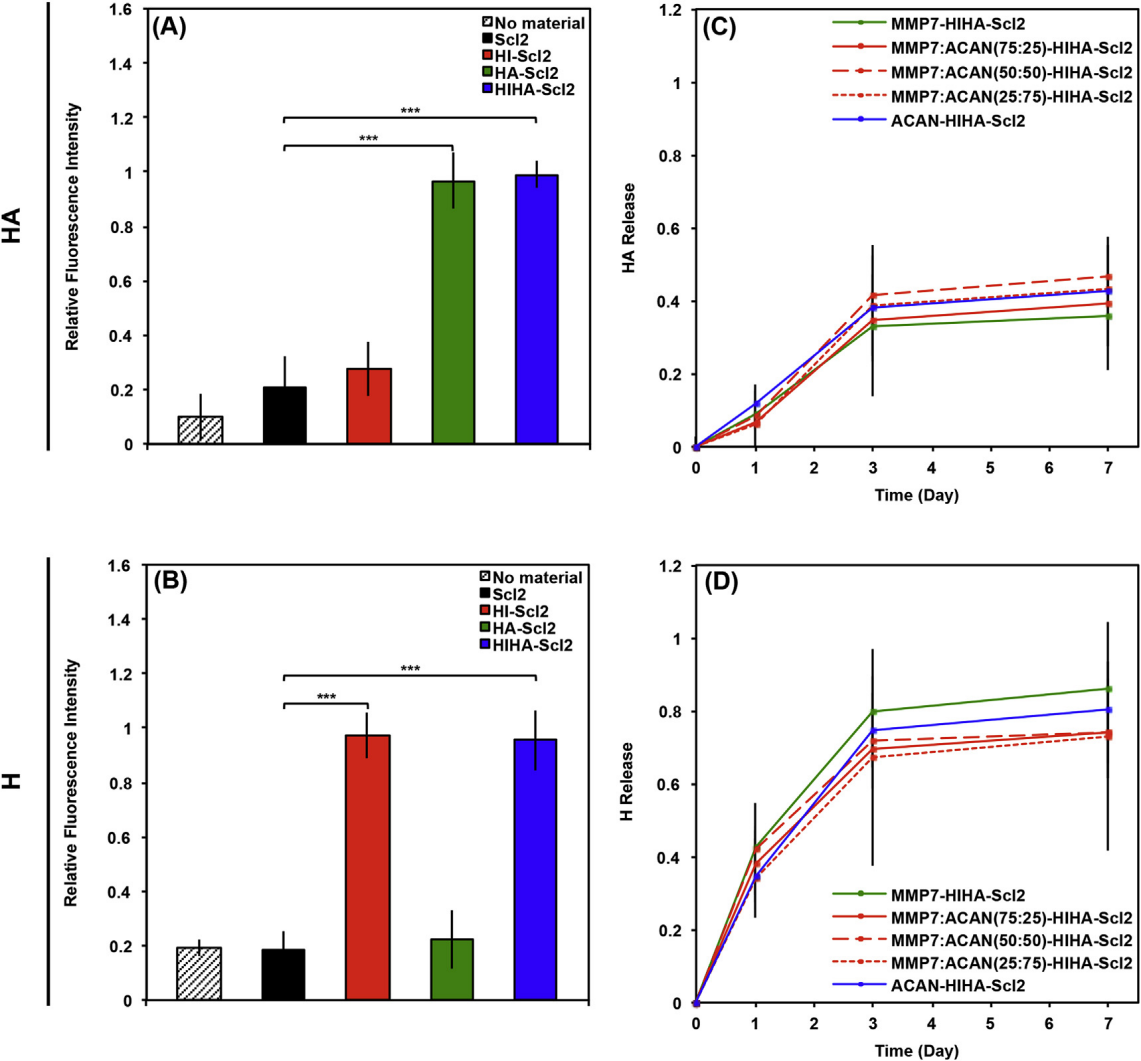
GAG binding on Scl2 hydrogels. (A–B) Binding of fluorescently–labeled (A) HA and (B) heparin to Scl2. Empty wells were used as negative control denoted ‘no material’. (C–D) Release of fluorescently–labeled (C) HA and (D) heparin from Scl2 hydrogels over 1 week. The fluorescence was measured in arbitrary units and relative binding of heparin or HA was normalized to the highest level of fluorescent intensity at each time point to provide the fraction of heparin or HA released over time. Values represent means ± SD. ***p < 0.001 (n = 3).

Scl2 proteins functionalized with HA–binding or H–binding peptide sequences displayed specific binding to HA or heparin (Fig. 3A–B), respectively. While there was some non–specific binding of HA and heparin to the HI-Scl2 and HA-Scl2 samples, this binding was not statistically significant when compared to the unmodified Scl2 proteins. Additionally, all hydrogel formulations showed a similar release of HA and heparin over time (Fig. 3C–D), likely due to the HA–binding and H–binding peptides increasing the overall charge of the hydrogels similarly leading to comparable binding with HA and heparin, respectively. This indicated that the different degradable peptides did not exhibit any specific interaction with HA or heparin. While both HA and heparin exhibited burst release characteristics over the first 3 days, possibly due to the hydrophilicity of the hydrogels, it is likely that binding and release of the GAGs from the hydrogels would be markedly different in the presence of cells or in the native environment and it would be a dynamic process.

### 3.2 Degradation kinetics

**Fig. 4:**
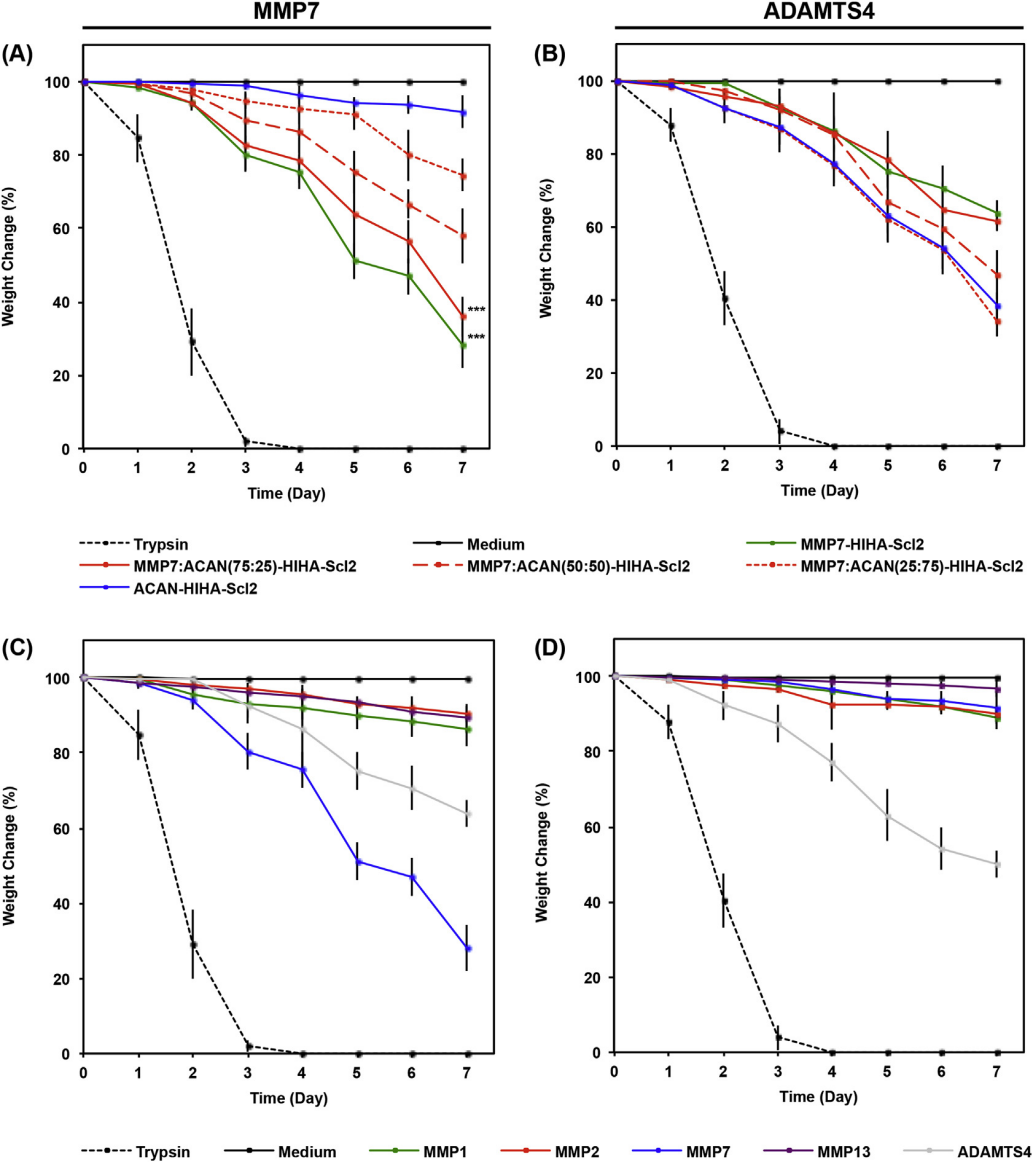
Degradation profile of acellular Scl2 hydrogels. Degradation of hydrogels incubated in chondrogenic medium supplemented with 30 ng/mL recombinant human (A) MMP7 or (B) ADAMTS4 over time characterized as dry weight loss and expressed as a percentage of initial dry weight. Degradation of hydrogels cross–linked via (C) MMP7 or (D) ADAMTS4 degradable peptides and exposed to 30 ng/mL recombinant human MMP1, 2, 7, or 13 over time characterized as dry weight loss and expressed as a percentage of initial dry weight. Trypsin–driven degradation is used as a positive control. Chondrogenic medium without exogenous enzymes is used as a negative control. Values represent means ± SD. ***p < 0.001 versus MMP7:ACAN(25:75)-HIHA-Scl2 (n = 3).

Fig. 4 demonstrates the degradation profile of acellular hydrogels in the presence of recombinant human MMP7 and ADAMTS4 compared to trypsin, MMP1, MMP2, and MMP13. Hydrogels cross–linked with the MMP7–cleavable peptide degraded much faster in the presence of MMP7 compared to ADAMTS4, MMP1, MMP2, and MMP13, for which cases less degradation was observed. However, ADAMTS4 degraded the MMP7–cleavable peptide to a greater extent than MMP1, MMP2, and MMP13, which could be expected because the MMP7–cleavable peptide sequence previously demonstrated some susceptibility to cleavage by aggrecanases [11], [21], [30], [31], [32]. This indicates that a complete breakdown of the hydrogel containing the MMP7–cleavable peptide cross–linker may still be possible in later stages of chondrogenesis. Hydrogels cross–linked with the ACAN–cleavable peptide degraded significantly more in the presence of ADAMTS4 compared to MMP1, MMP2, MMP7, and MMP13, in which cases minimal degradation was observed. This highlights the higher specificity of the ACAN–cleavable peptide to ADAMTS4 compared to the MMP7–cleavable peptide, which is susceptible to degradation by both MMP7 and to a lesser extent ADAMTS4. The higher specificity of the ACAN–cleavable peptide compared to the MMP7–cleavable peptide may be advantageous *in vivo* because it suggests that ACAN–cleavable peptide would hold the hydrogel structure together to support the hMSCs until mature chondrocytes are formed. Some non–specific degradation of all hydrogels by MMP1, MMP2, and MMP13 was observed, likely because MMPs are known to recognize and cleave a range of peptide sequences [4], [11], [24], [30], [33]. The combination of the MMP7– and ACAN–cleavable peptide cross–linkers led to similar degradation rates among all hydrogel samples in the presence of ADAMTS4 compared to hydrogel samples in the presence of MMP7, again demonstrating some ability of ADAMTS4 to also degrade the MMP7–cleavable peptide.

### 3.3 hMSC behavior in bioactive Scl2 hydrogels

#### 3.3.1 Cell adhesion and viability

**Fig. 5:**
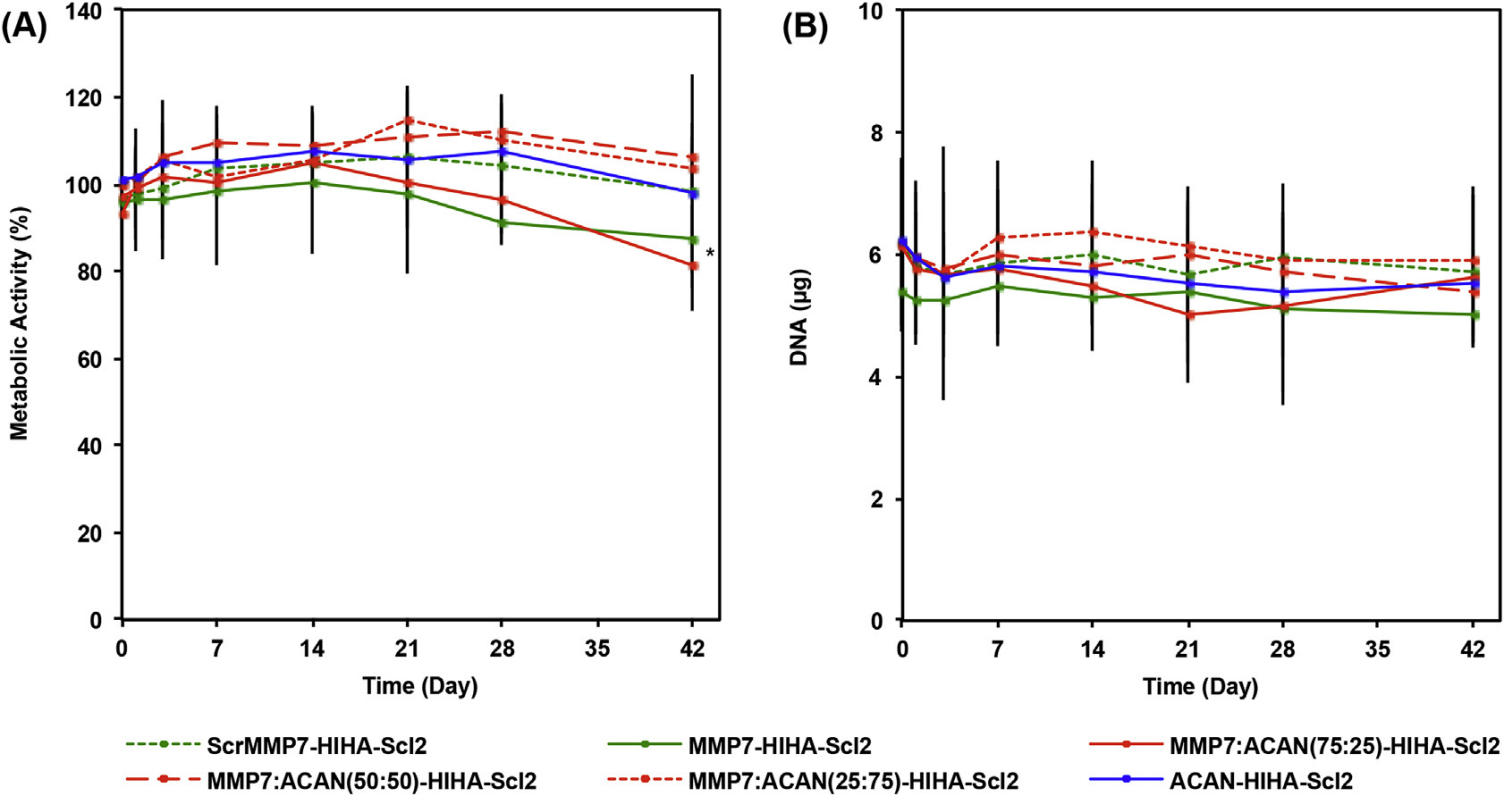
Metabolic activity and DNA content of hMSCs cultured in Scl2 hydrogels. (A) hMSC metabolic activity in hydrogels over 6 weeks in culture. (B) DNA content of hMSCs per construct in hydrogels cultured over 6 weeks in culture. All data normalized to day 0. Values represent means ± SD. *p < 0.05 versus MMP7:ACAN(25:75)-HIHA-Scl2 (n = 3 for each donor; 3 different bone marrow–derived hMSC donors).

The metabolic activity of encapsulated hMSCs and DNA content were maintained at consistently high levels for all hydrogel formulations that only differed in the type of peptide cross–linker throughout the culture period (Fig. 5). The long–term viability of encapsulated hMSCs in all hydrogels was further confirmed qualitatively via a LIVE/DEAD^®^ assay at day 42 (Fig. S6A–F). The effectiveness of the I-binding peptide sequence incorporated into the Scl2 backbone, previously shown to bind to the α1β1 and α2β1 integrins [15], was also validated (Fig. S7). These results suggest that the combination of the H–binding, HA–binding, and I–binding motifs on the Scl2 protein is sufficient to promote long–term cell viability most likely through direct cell binding and ECM binding interactions. The presence of the H–binding, I–binding, and HA–binding peptides have previously been shown to aid in maintaining long–term cell viability and in impacting biological processes through their interaction with heparin and HA [11], [15]. It should be mentioned that the metabolic activity of hMSCs in the MMP7-HIHA-Scl2 and MMP7:ACAN(75:25)-HIHA-Scl2 hydrogels was significantly lower compared to the other hydrogel formulations at day 42. This is likely because these hydrogels with the greatest proportion of MMP7 peptide are likely to degrade faster, possibly decreasing matrix accumulation.

#### 3.3.2 In vitro chondrogenesis of hMSCs in degradable Scl2 hydrogels

**Fig. 6:**
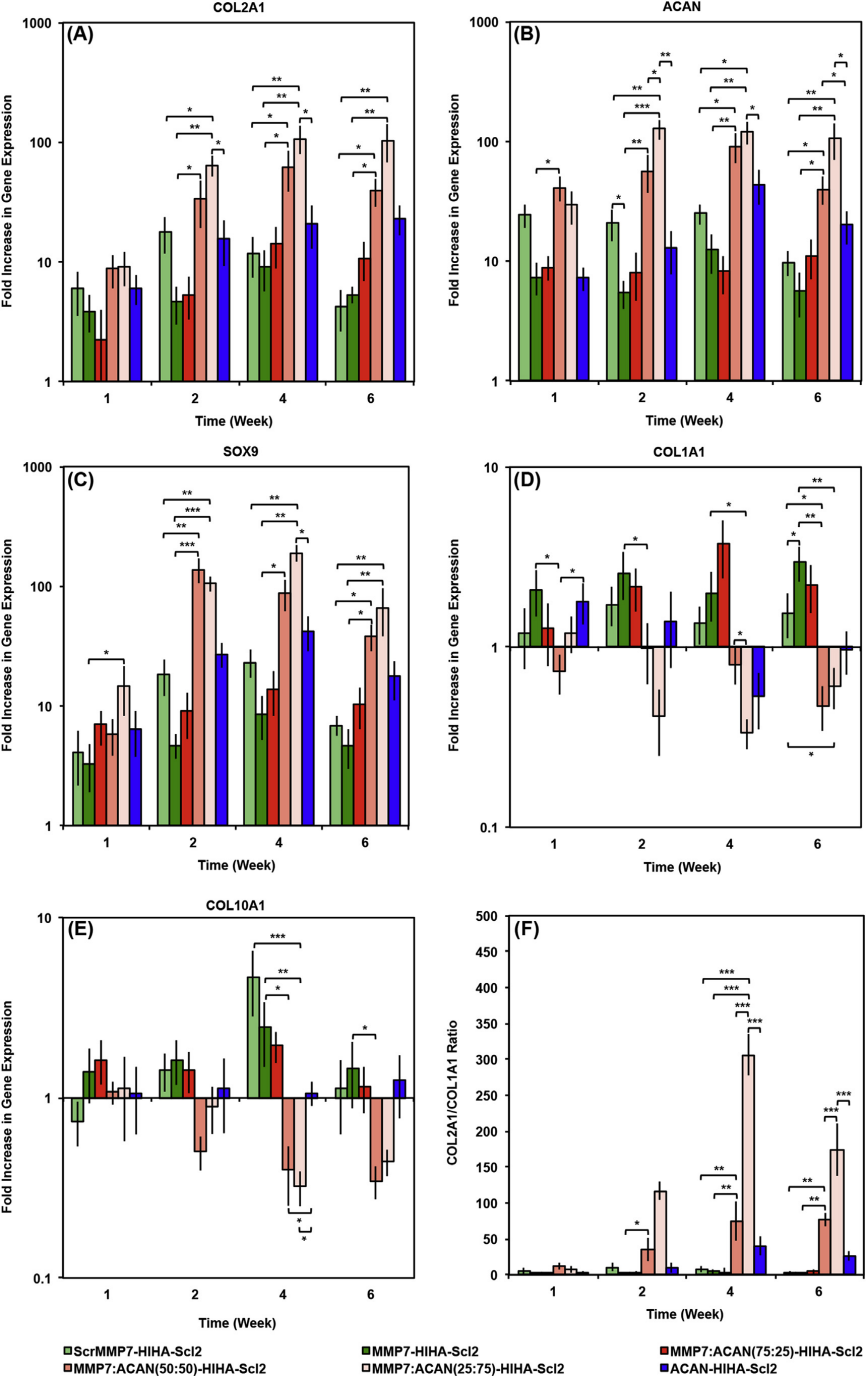
hMSC gene expression in Scl2 hydrogels. (A) COL2A1, (B) ACAN, (C) SOX9, (D) COL1A1, and (E) COL10A1 gene expression of hMSCs encapsulated in hydrogels over 6 weeks in culture, as analyzed using the ΔΔCt method. Data presented as a fold difference relative to undifferentiated hMSCs (calibrator) prior to encapsulation and normalized to GAPDH (housekeeping gene). (F) COL2A1/COL1A1 gene expression ratio. Values represent means ± SD. *p < 0.05, **p < 0.01, ***p < 0.001 (n = 3 for each donor; 3 different bone marrow–derived hMSC donors).

The effects of the MMP7– and ACAN–cleavable peptide cross–linkers used alone or in combination on the chondrogenic differentiation of encapsulated hMSCs were compared to the non–degradable ScrMMP7 peptide cross–linker control [11], [21], [22], [30], [34]. The gene expression of chondrogenic markers (COL2A1, ACAN, and SOX9) (Fig. 6A–C) all suggested that the MMP7:ACAN(50:50)-HIHA-Scl2 and MMP7:ACAN(25:75)-HIHA-Scl2 hydrogels, which are the hydrogel formulations containing a combination of the degradable peptides, promoted chondrogenesis of hMSCs to the greatest extent from week 2 to week 6. This result suggests a unique and powerful synergistic effect due specifically to the concurrent presentation of two distinct degradable peptide motifs. Furthermore, the gene expression levels of chondrogenic markers in our Scl2 hydrogels, particularly in the hydrogels with dual peptides, are improved compared to the differentiation of hMSCs in a pellet culture system at 2 weeks (Fig. S8) and pellet culture systems reported in the literature [35]. Similarly interesting, hMSCs in the ACAN-HIHA-Scl2 hydrogels had significantly higher COL2A1, ACAN, and SOX9 gene expression from week 2 to week 6 compared to the MMP7-HIHA-Scl2 hydrogels. This suggests that the ACAN–cleavable peptide, which has not yet been explored for tissue engineering applications with hMSCs, may have promoted chondrogenesis on its own. However, this effect could also be due to the degradation rate of the hydrogel at a specific time point or by diverting ADAMTS4 to degrade the hydrogel rather than the deposited matrix, meriting further exploration of this peptide for cartilage tissue engineering applications.

Another unexpected observation was that hMSCs in the ScrMMP7-HIHA-Scl2 hydrogels had higher gene expression of ACAN and SOX9 at all time points compared to those in the MMP7-HIHA-Scl2 hydrogels that reflected statistical reality. This is likely because the MMP7-HIHA-Scl2 hydrogels degraded at a faster rate compared to the ScrMMP7-HIHA-Scl2 hydrogels that are engineered to be non–degradable, resulting in less matrix accumulation and thereby lesser impact on cell behavior. This highlights the complexity involved in tuning the biodegradability of biomaterials for optimal tissue regeneration. In accordance with our previous work, it is not surprising that all hydrogels exhibited enhanced chondrogenic marker gene expression profiles compared to undifferentiated hMSCs [11]. This is likely because the HA–binding and H–binding peptides mimic protein–GAG interactions in the native environment [22], thus validating the use of the modified Scl2 protein for cartilage tissue engineering purposes. Additionally, heparin is present in native articular cartilage and it is known to recruit endogenous growth factors such as TGF–β, BMPs, and bFGF, and form stable complexes that protect against proteolytic degradation [36], [37]. This can result in enhanced biological activity and delayed growth factor release, which is considered important because growth factors known to facilitate chondrogenesis such as TGF–β have a relatively short half–life. Thus, prolonging their stability and possibly enhancing their biological activity in long–term culture could indirectly promote chondrogenesis. Previous studies have shown the presence of the RGD peptide sequence to be important during early stage hMSC chondrogenesis [4]. However, its persistence in hydrogel systems has been shown to delay or inhibit complete chondrogenesis. It is possible that the I-binding peptide had a similar effect to the RGD peptide on hMSC chondrogenesis. In contrast to gene expression profiles of chondrogenic markers, hMSC gene expression levels of osteogenic (Runx2) and lipogenic (PPAR-γ) markers were down–regulated at week 2 and thus, further confirming that the hMSCs had committed towards the chondrogenic lineage in our hydrogels (Fig. S8). Quantification of immunohistochemical staining for chondrogenic, osteogenic, and lipogenic markers as an indicator of hMSC population heterogeneity further demonstrated that by 2 weeks, at least 60% of hMSCs were committed to chondrogenesis in all hydrogels (Fig. S8).

Gene expression levels of COL1A1 by hMSCs were significantly elevated for the non–degradable control and the conditions with the greatest fraction of the MMP7–cleavable peptide at week 6, including ScrMMP7-HIHA-Scl2 and MMP7:ACAN(75:25)-HIHA-Scl2 hydrogels (Fig. 6D). COL1A1 is the major component of fibrocartilage, which is characteristic of inadequate articular cartilage healing and provides inferior mechanical properties compared to collagen type II [38], [39]. In addition, the hMSC gene expression levels of COL10A1 were significantly higher in MMP7-HIHA-Scl2 hydrogels at week 6 (Fig. 6E). Elevated COL10A1 is characteristic of chondrocyte hypertrophy [40]. Thus, increased COL1A1 and COL10A1 gene expression in the MMP7 peptide–rich hydrogels indicated progress towards terminal differentiation of hMSCs and a hypertrophic phenotype, which is a critical complication for the regeneration of articular cartilage tissue and for the potential use of hMSCs in the clinic [11], [41], [42]. As such, the inhibition of hypertrophic marker gene expression by the ACAN–cleavable peptide alone and to a greater extent in combination with the MMP7–cleavable peptide is highly promising, and consistent with previous work demonstrating that collagen type I and collagen type X deposition by chondrocytes were decreased in hydrogels with the ACAN–cleavable peptide cross–linked compared to non–degradable hydrogels [21]. This result demonstrates that the previously observed improvements in cartilaginous matrix quality by chondrocytes can be translated to hMSCs undergoing chondrogenic differentiation. The COL2A1/COL1A1 gene expression ratio, a measure of the differentiation index [43], remained significantly higher for the MMP7:ACAN(25:75)-HIHA-Scl2 hydrogels compared to the other hydrogel formulations throughout the culture period (Fig. 6F). This implies that this specific combination of enzyme–cleavable peptides with a larger fraction of the ACAN–cleavable peptide maintained chondrogenic marker gene expression to the greatest extent over 6 weeks of culture.

**Fig. 7:**
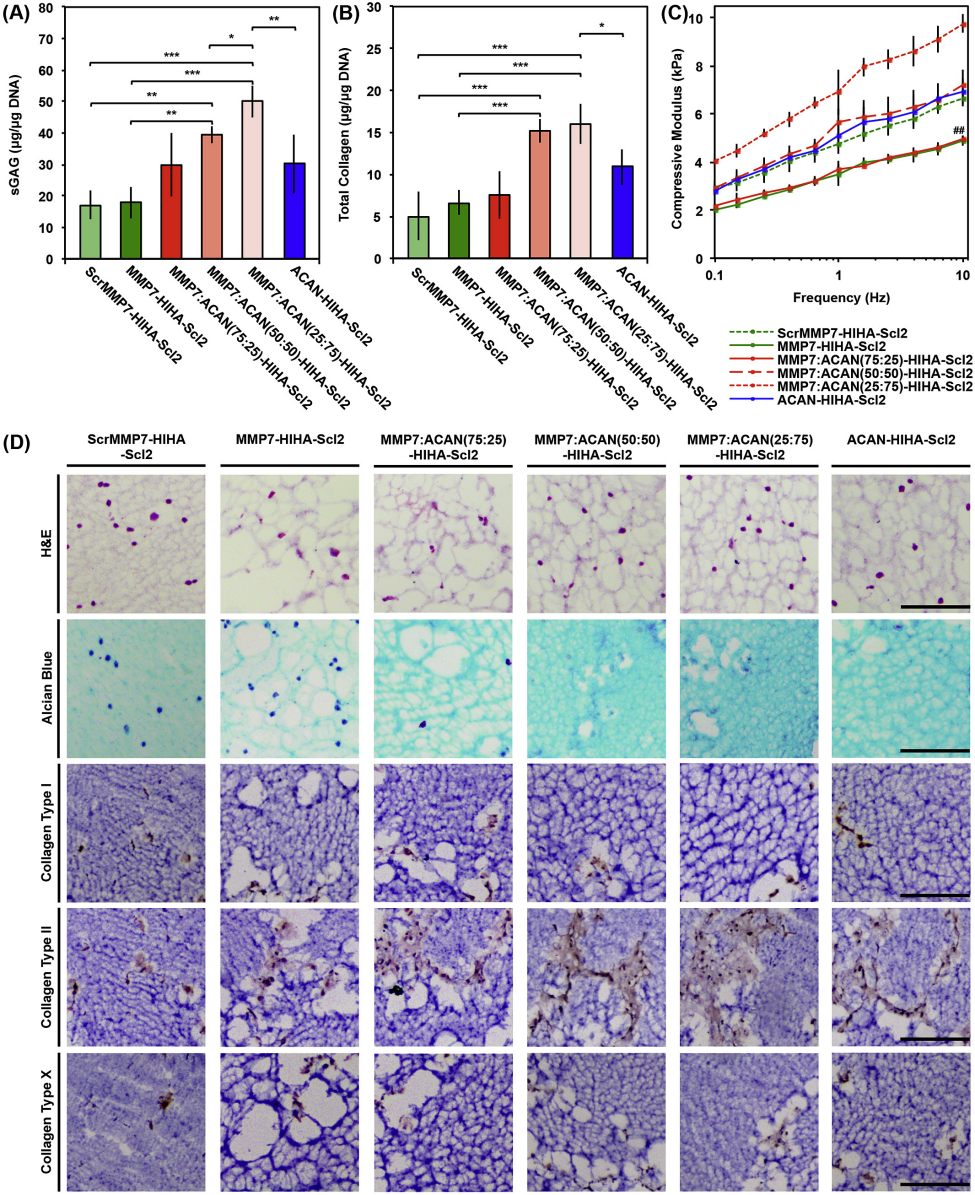
ECM accumulation and compressive modulus of hMSC–seeded Scl2 hydrogels. (A) Sulfated glycosaminoglycan (sGAG) content of tissue deposited by hMSCs in hydrogels over 6 weeks in culture. (B) Hydroxyproline content of tissue deposited by hMSCs in hydrogels over 6 weeks in culture as an estimation of total collagen content. (C) Dynamic mechanical analysis (DMA) used to determine the elastic modulus of compression of hMSC–seeded hydrogels compressed to 10% strain at 0.5% strain/min and 0.1–10 Hz after 6 weeks in culture. Values represent means ± SD. *p < 0.05, **p < 0.01, ##*p* < 0.01 versus MMP7:ACAN(25:75)-HIHA-Scl2, ***p < 0.001 (n = 3 for each donor; 3 different bone marrow–derived hMSC donors). (D) Representative histological and immunohistochemical examination of hMSC–seeded hydrogels after 6 weeks in culture. Hydrogels are stained with Haematoxylin and Eosin (H&E), Alcian Blue for sGAG, and by immunohistochemistry for collagen type I, collagen type II, and collagen type X, respectively, from top to bottom. Scale bars are 50 μm.

The hMSC–seeded hydrogels cultured over 6 weeks were assessed for sGAG and collagen accumulation to further examine the extent of chondrogenic differentiation, where cartilage–specific matrix is predominantly characterized by sGAG and collagen deposition [44]. sGAG content normalized to DNA (Fig. 7A) was significantly greater for the MMP7:ACAN(25:75)-HIHA-Scl2 hydrogels compared to all other hydrogel formulations at week 6. Additionally, the total collagen content normalized to DNA (Fig. 7B) was significantly higher for the combined peptide conditions, MMP7:ACAN(50:50)-HIHA-Scl2 and MMP7:ACAN(25:75)-HIHA-Scl2, compared to all other samples at week 6. These combined peptide conditions with highest matrix accumulation further translated into significantly higher compression moduli (Fig. 7C and Fig. S9), which was further supported by their minimal construct weight loss over time (Fig. S10). Moreover, the compression moduli of the cell–seeded hydrogels increased slightly compared to acellular hydrogels during *in vitro* culture, which is an interesting result because it is generally more common for degradable hydrogel systems to exhibit a loss in compression moduli. The compression moduli of the hydrogels is also expected to demonstrate greater improvements in an *in vivo* setting for treating focal defects in articular cartilage, and thus potentially facilitating implantation and the long–term performance [45].

Histological examination of the hydrogels indicated a sparse distribution of cells and extensive matrix accumulation at day 42 compared to day 0, day 14, and day 21 (Fig. 7D, Fig. S11–S13). More dispersed sGAG accumulation (Alcian Blue staining) was observed in the MMP7:ACAN(50:50)-HIHA-Scl2 and MMP7:ACAN(25:75)-HIHA-Scl2 hydrogels compared to the MMP7-HIHA-Scl2 and ScrMMP7-HIHA-Scl2 samples, which was supported by the higher sGAG accumulation results. Immunohistochemical staining for collagen type II was noticeably increased compared to collagen type I and collagen type X deposition in all hydrogels, indicating a general promotion of the cartilage–specific phenotype for all hydrogels [41], [44]. The immunohistochemical images indicated that at 6 weeks, levels of new matrix deposition were still far from sufficient to create a functional cartilaginous construct, however, the matrix produced in all degradable constructs was of enhanced quality compared to the non–degradable control, which qualitatively exhibited the least matrix deposition. It is also possible that mechanical stimulation of the constructs may help to enhance matrix production by better simulating the native environment.

#### 3.3.3 Temporal expression and activity of proteases

**Fig. 8:**
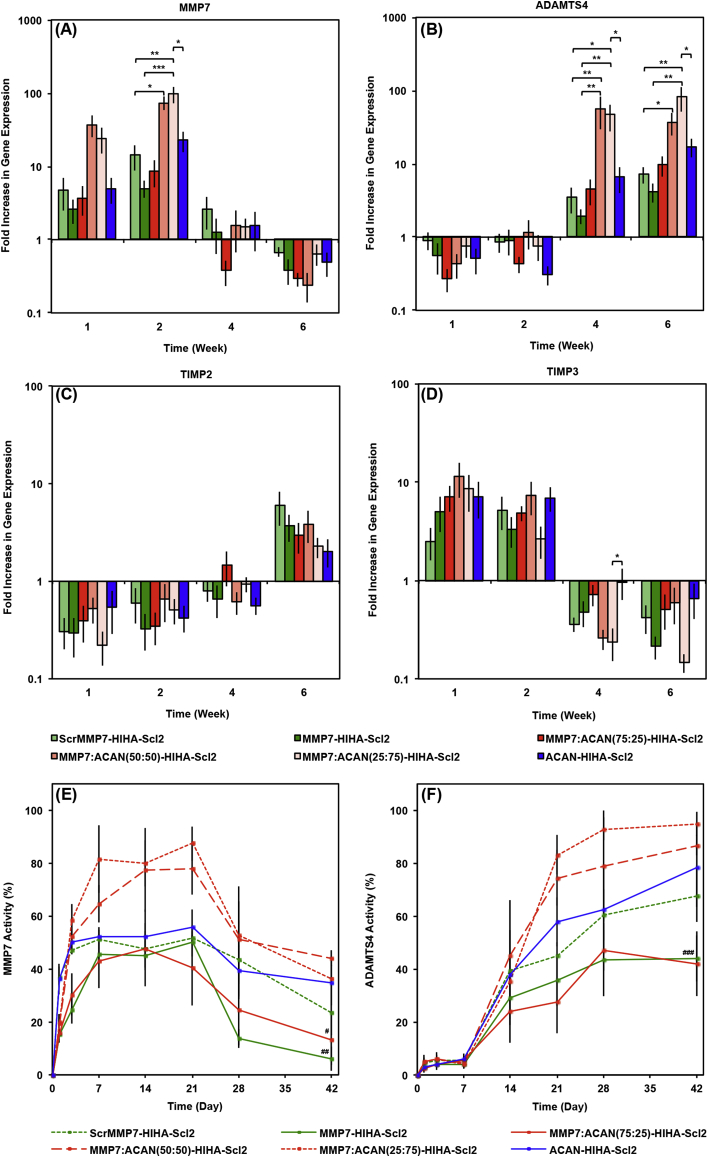
hMSC gene expression and enzymatic activity in Scl2 hydrogels. (A) MMP7, (B) ADAMTS4, (C) TIMP2, and (D) TIMP3 gene expression of hMSCs encapsulated in hydrogels over 6 weeks in culture, as analyzed using the ΔΔCt method. Data presented as a fold difference relative to undifferentiated hMSCs (calibrator) prior to encapsulation and normalized to GAPDH (housekeeping gene). Activity of (E) MMP7 and (F) ADAMTS4 in hydrogels over 6 weeks in culture. MMP7 and ADAMTS4 activities were normalized to fluorescence signal output at day 0. Values represent means ± SD. *p < 0.05, ^#^*p* < 0.05 versus MMP7:ACAN(25:75)-HIHA-Scl2, **p < 0.01, ^##^*p* < 0.01 versus MMP7:ACAN(25:75)-HIHA-Scl2, ***p < 0.001, ^###^*p* < 0.001 versus MMP7:ACAN(25:75)-HIHA-Scl2 (n = 3 for each donor; 3 different bone marrow–derived hMSC donors).

As matrix remodeling is closely associated with chondrogenesis [46], and the hydrogels were specifically designed to respond to the temporal expression of matrix remodeling enzymes, it was important to confirm the gene expression and activity of the targeted proteases over the 6 week study. Overall, the gene expression and activity of MMP7 were highest from week 1 to week 3, followed by elevated ADAMTS4 gene expression and activity from week 3 to week 6 (Fig. 8), validating the different temporal expression profiles of these enzymes during the process of chondrogenesis. This data supports our hypothesis that both enzymes are expressed during the process of chondrogenesis, where MMP7 is expressed initially and ADAMTS4 is expressed later following commitment to the chondrocyte lineage [19], [24]. Because there was minimal overlap in the time–course of enzyme activity, it can be assumed that early degradation was targeted specifically to the MMP7–cleavable peptide, and later stage degradation would be predominantly targeted to the ACAN–cleavable peptide. It is very difficult to de–couple hydrogel degradation from matrix accumulation and potential biological signaling effects in a reliable and robust manner. However, the dry weight change of acellular and cell–laden hydrogel constructs, alongside the analysis of acellular degradation characteristics and enzymatic gene expression and activity levels, can help provide an insight into the combined effect. During the early stages of culture, where MMP7 activity was highest, the construct weight decrease (Fig. S10) was significantly more drastic for the MMP7-HIHA-Scl2 and MMP7:ACAN(75:25)-HIHA-Scl2 hydrogels, indicating that degradation was mostly mediated via endogenous MMP7 rather than ADAMTS4. Because some ADAMTS4–mediated degradation was observed for the MMP7–cleavable peptide (Fig. 4B), we can also infer that later–stage ADAMTS4 activity could also continue degrading any remaining MMP7 peptide, which would be desirable in leading to complete scaffold degradation over time.

Looking more closely at the results, the gene expression levels of MMP7 were significantly elevated in hMSCs encapsulated within the MMP7:ACAN(25:75)-HIHA-Scl2 hydrogels compared to the MMP7-HIHA-Scl2 and ACAN-HIHA-Scl2 hydrogels from week 1 to week 2 (Fig. 8A). Furthermore, the gene expression of ADAMTS4 was significantly greater in the same combined peptide hydrogel formulation at week 6 (Fig. 8B). Since this was the condition exhibiting the most chondrogenesis–specific gene markers and highest cartilaginous matrix production, this also suggested that elevated enzyme–mediated scaffold degradation may be beneficial to the process of chondrogenesis and stimulating for matrix production. Additionally, high levels of ADAMTS4 gene expression are not observed in undifferentiated hMSCs [24], [31], [47], and high levels of MMP7 gene expression are not known to be found in chondrocytes [24], [30]. Therefore, our results suggest a transition from an undifferentiated hMSC to mature chondrocyte phenotype after 3–4 weeks of culture in the bioactive Scl2 hydrogels. The hMSC gene expression of TIMP2, a known inhibitor of MMP7 [48], exhibited a reverse trend to MMP7 gene expression in all hydrogels with the lowest levels present at week 1 and week 2 (Fig. 8C). Similarly, the hMSC gene expression of TIMP3, a known inhibitor of ADAMTS4 [31], [32], [47], was reverse–correlated with the ADAMTS4 gene expression in all hydrogels, with the lowest levels measured at week 4 and week 6 (Fig. 8D). In addition, heparin is a highly sulfated GAG that is thought to promote the autolytic molecular activation of proMMP7 and activity of MMP7 [30] as well as the activity of ADAMTS4 [49]. These results further reveal an important switch in cellular regulation processes during the 6 week culture period in our novel bioactive hydrogels.

The compression moduli of the MMP7:ACAN(25:75)-HIHA-Scl2 hydrogels increased significantly compared to the acellular counterpart after 6 weeks (*P* < 0.01). Our results showed that these combined cleavable peptide hydrogels accumulated a significantly greater amount of sGAG and total collagen as well as exhibiting minimal construct weight loss compared to the MMP7-HIHA-Scl2 and MMP7:ACAN(25:75)-HIHA-Scl2 hydrogels, which likely contributed to the improved compression moduli of the hydrogels. After week 4, the construct weight change for the MMP7:ACAN(50:50)-HIHA-Scl2 and MMP7:ACAN(25:75)-HIHA-Scl2 hydrogels plateaued and increased for the latter compared to the MMP7-HIHA-Scl2 and MMP7:ACAN(75:25)-HIHA-Scl2 hydrogels, suggesting a balance between the degradation of the hydrogel and the accumulation of ECM, which is supported by the increased sGAG and collagen deposition. This type of balance between scaffold degradation and matrix production was predicted by computational modeling simulations as the most optimal pattern to improve cartilage tissue engineering outcomes when implementing degradable scaffolds, and also points towards a more localized as opposed to bulk degradation mode [50]. The ability to achieve scaffold degradation while maintaining overall mechanical integrity has been noted as a major advantage for enzymatically degradable systems as opposed to more passive, bulk degrading systems [21], [51], [52]. Moreover, it may be possible to conduct studies in an attempt to de–couple hydrogel degradation and matrix accumulation by observing the temporal biodegradability of hydrogels through radio–labeling of the peptide cross–linkers, and these types of experiments could be considered for future studies to further assess the implications of temporal biodegradability. Additionally, the MMP7-HIHA-Scl2 and MMP7:ACAN(75:25)-HIHA-Scl2 hydrogels swelled significantly more compared to the MMP7:ACAN(50:50)-HIHA-Scl2 and MMP7:ACAN(25:75)-HIHA-Scl2 hydrogels after 6 weeks (Fig. S14), agreeing with the lower compression moduli of the hydrogels containing a higher fraction of the MMP7–cleavable peptide cross–linker compared to the combined peptide cross–linker hydrogels. It is known that the swelling behavior of native tissues is closely linked to their compression moduli [53]. Hydrogels cross–linked via the combination of MMP7– and ACAN–cleavable peptides have demonstrated cell–mediated degradation that encourages chondrogenesis and differentiation to a greater degree than either peptide used alone [4], [11], [24], [54], [55], [56], and in the current work have been shown to maintain construct mechanical integrity as well as a balance between scaffold degradation and matrix accumulation. The use of the MMP7– and ACAN–cleavable peptides as cross–linkers within a single hydrogel system is shown to facilitate complex, temporal degradation patterns that match the chondrogenesis of encapsulated hMSCs, enabling accumulation and evolution of cartilage–specific matrix. To the best of our knowledge, this study represents the first demonstration of a bimodal enzymatically degradable hydrogel, where the hydrogel design was tailored to different temporal expression patterns of enzymes produced during the chondrogenesis of hMSCs. The positive synergistic effect from combining two distinct degradable peptides within one hydrogel system is highly promising, meriting further investigation into multimodal degradation approaches.

The successful use of multiple enzymatically–cleavable peptides in combination to tune hydrogel degradability and with a variety of bioactive moieties highlights the possibility to facilitate complex biological processes and more closely mimic the dynamics and complexity of native tissue repair, regeneration, and remodeling. Taking advantage of the modularity of our system, it is possible to introduce greater complexity and fine tuning to specific cellular processes through the incorporation of other enzyme–sensitive and bioactive peptide sequences via tethering or site–directed mutagenesis to the Scl2 backbone. These key features of our hydrogel platform can be especially advantageous for future clinical translation as it enables the ability to recapitulate a high degree of biological complexity in a controlled system, which could enhance biorecognition by native cells and matrix molecules. Our novel Scl2 system provides a useful platform that can be easily adapted for other applications in regenerative medicine and tissue engineering.

## 4 Conclusions

In this work, we designed and manufactured novel collagen–mimetic hydrogels that can be specifically modified and tailored to recapitulate features of the complex and dynamic ECM of articular cartilage. We modified the backbone of ‘blank slate’ Scl2 proteins with H–binding, I–binding, and HA–binding sequences through site–directed mutagenesis. The proteins were cross–linked into injectable hydrogels via a combination of MMP7 and ADAMTS4–cleavable peptides at varying ratios for cell–mediated degradation tuned to the temporal expression of catabolic enzymes during and following chondrogenic differentiation of encapsulated hMSCs. The combination of the MMP7 and ADAMTS4–degradable cross–linkers significantly enhanced the chondrogenesis of hMSCs compared to the use of either peptide cross–linker alone. Specifically, the MMP7:ACAN(50:50)-HIHA-Scl2 and MMP7:ACAN(25:75)-HIHA-Scl2 hydrogels significantly up–regulated chondrogenic marker gene expression by hMSCs resulting in the highest sGAG and collagen accumulation and maintenance of mechanical integrity over the culture period, which overcomes major challenges inherent to degradable constructs for tissue engineering cartilage. These results suggest a balance between the degradation of the hydrogels and the accumulation of matrix by hMSCs, which is an important result that also signifies the necessity to design more complex degradable constructs that are responsive to temporal changes in cellular metabolism. Our highly versatile hydrogels demonstrate the potential to facilitate specific biological processes to mimic the dynamics and complexity of native tissues, which could ultimately be translated into a minimally invasive clinical approach to deliver autologous hMSCs for cartilage regeneration therapies.
