# Supplementary material for: Temporally degradable collagen–mimetic hydrogels tuned to chondrogenesis of human mesenchymal stem cells
Source: Biomaterials. 2016 Aug;99:56–71. doi: 10.1016/j.biomaterials.2016.05.011 (PMC4910873; doi:10.1016/j.biomaterials.2016.05.011)
Supplement: Supplementary file 1 [file mmc1.pdf]

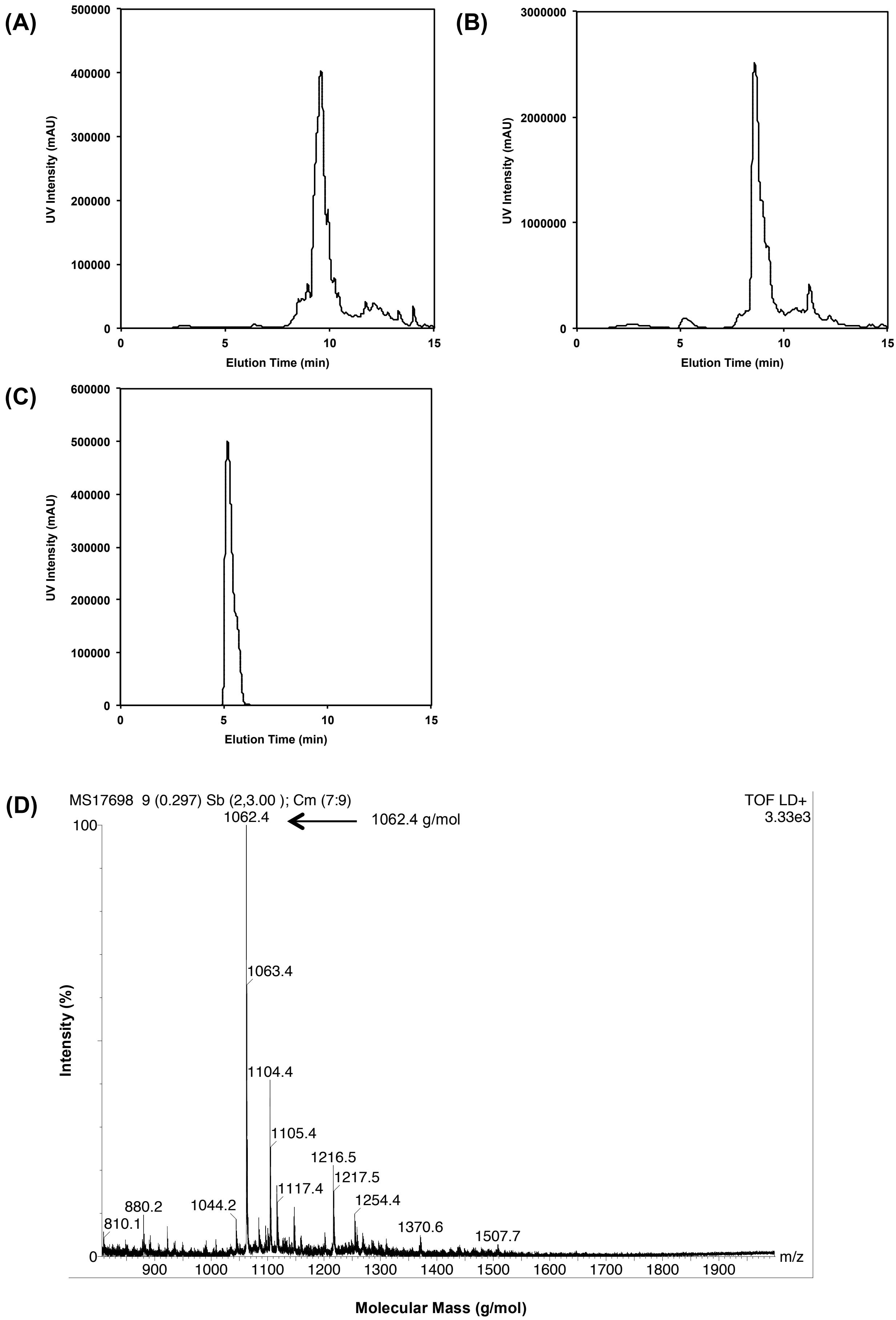
(A)
UV Intensity (mAU)
Elution Time (min)
0
100000
200000
300000
400000
500000
0
5
10
15
(B)
UV Intensity (mAU)
Elution Time (min)
0
1000000
2000000
3000000
0
5
10
15
(C)
UV Intensity (mAU)
Elution Time (min)
0
100000
200000
300000
400000
500000
600000
0
5
10
15
(D)
MS17698 9 (0.297) Sb (2,3.00); Cm (7:9)
TOF LD+
3.33e3
1062.4
1062.4 g/mol
1063.4
1104.4
1105.4
1216.5
1217.5
1117.4
1254.4
880.2
1044.2
810.1
1370.6
1507.7
100
0
900
1000
1100
1200
1300
1400
1500
1600
1700
1800
1900
m/z
Intensity (%)
Molecular Mass (g/mol)

(E)

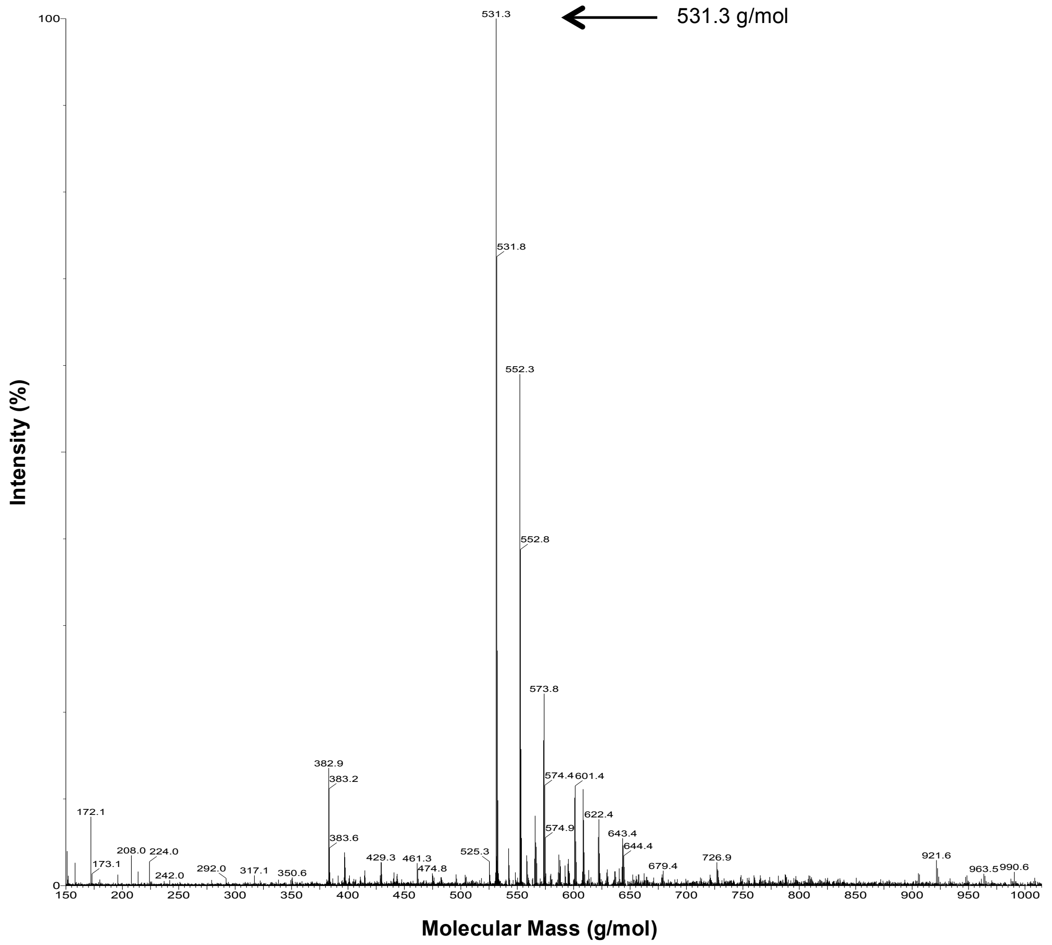

(F)

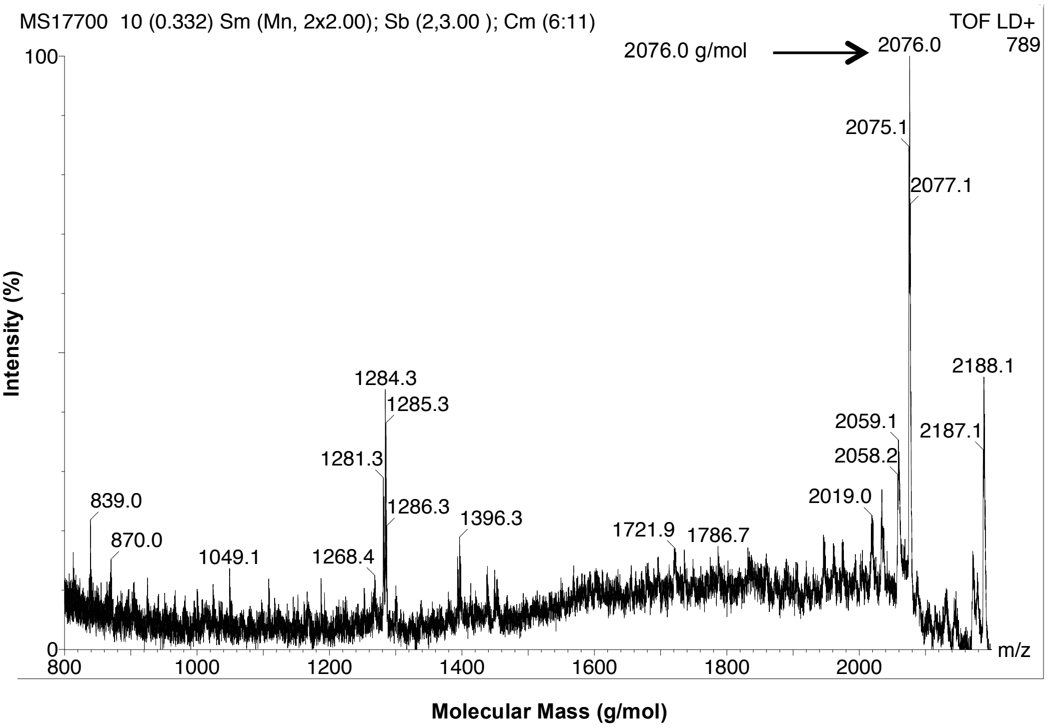

Fig. S1. Representative reverse phase HPLC chromatograms and MALDI/ESI spectra of (A, D) MMP7 (MW: 1062.4 g/mol), (B, E) ScrMMP7 (MW/2: 531.3 g/mol, MW: 1062.6 g/mol), and (C, F) ACAN (MW: 2076.0 g/mol) peptides, respectively.

(A)

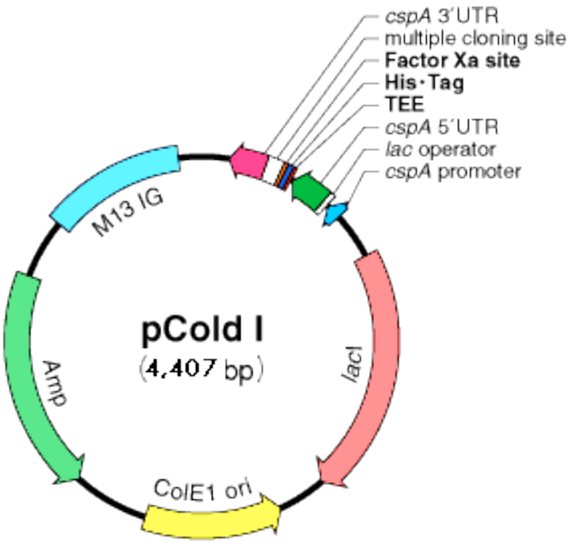


```
pCold I vector sequence:
   1  AAGGAATGGT GTGGCCGATT AATCATAAAT ATGAAAAATA ATTGTTGCAT CACCCGCCAA TGCGTGGCTT AATGCACATC AAATTGTGAG CGGATAACAA

              BmtI
            ------+
              NheI
            -+-----
 101  TTTGATGTGC TAGCGCATAT CCAGTGTAGT AAGGCAAGTC CCTTCAAGAG TTATCGTTGA TACCCCTCGT AGTGCACATT CCTTTAACGC TTCAAAATCT

 201  GTAAAGCACG CCATATCGCC GAAAGGCACA CTTAATTATT AAGAGGTAAT ACACCATGAA TCACAAAGTG CATCATCATC ATCATCATAT CGAAGGTAGG

                         AbsI
                       --+------
                  KpnI                                  PstI
                 -----+                                ------+
            SacI         PspXI                 AccI
           ------+     --+------              --+---
           Eco53kI       XhoI         EcoRI    SalI
           ---+---      -+-----      -+----   -+----
      NdeI        Acc65I       BamHI       HindIII   BspMI    XbaI
      --+---     -+----      -+----      -+------  -------  -+----
 301  CATATGGAGC TCGGTACCCT CGAGGGATCC GAATTCAAGC TTGTCGACCT GCAGTCTAGA TAGGTAATCT CTGCTTAAAA GCACAGAATC TAAGATCCCT

                                                ClaI
                                               --+---
 401  GCCATTTGGC GGGGATTTTT TTATTTGTTT TCAGGAAATA AATAATCGAT CGCGTAATAA AATCTATTAT TATTTTTGTG AAGAATAAAT TTGGGTGCAA

         BsmI   Eco0109I                                   NspI
        ------  --+----                                   -----+
 501  TGAGAATGCG CAGGCCCTTT CGTCTCGCGC GTTTCGGTGA TGACGGTGAA AACCTCTGAC ACATGCAGCT CCCGGAGACG GTCACAGCTT GTCTGTAAGC

 601  GGATGCCGGG AGCAGACAAG CCCGTCAGGG CGCGTCAGCG GGTGTTGGCG GGTGTCGGGG CTGGCTTAAC TATGCGGCAT CAGAGCAGAT TGTACTGAGA

 701  GTGCACCATA AAATTGTAAA CGTTAATATT TTGTTAAAAT TCGCGTTAAA TTTTTGTTAA ATCAGCTCAT TTTTTAACCA ATAGGCCGAA ATCGGCAAAA

         PsiI
        ---+--
 801  TCCCTTATAA ATCAAAAGAA TAGCCCGAGA TAGGGTTGAG TGTTGTTCCA GTTTGGAACA AGAGTCCACT ATTAAAGAAC GTGGACTCCA ACGTCAAAGG

                                   BsaAI
                                  ---+--
 901  GCGAAAAACC GTCTATCAGG GCGATGGCCC ACTACGTGAA CCATCACCCA AATCAAGTTT TTTGGGGTCG AGGTGCCGTA AAGCACTAAA TCGGAACCCT

                                      NaeI
                                    ---+---
                                     NgoMIV
                                    -+-----
1001  AAAGGGAGCC CCCGATTTAG AGCTTGACGG GGAAAGCCGG CGAACGTGGC GAGAAAGGAA GGGAAGAAAG CGAAAGGAGC GGGCGCTAGG GCGCTGGCAA

1101  GTGTAGCGGT CACGCTGCGC GTAACCACCA CACCCGCCGC GCTTAATGCG CCGCTACAGG GCGCGTACTA TGGTTGCTTT GACGTATGCG GTGTGAAATA

1201  CCGCACAGAT GCGTAAGGAG AAAATACCGC ATCAGGCGTC AGGTGGCACT TTTCGGGGAA ATGTGCGCGG AACCCCTATT TGTTTATTTT TCTAAATACA

1301  TTCAAATATG TATCCGCTCA TGAGACAATA ACCCTGATAA ATGCTTCAAT AATATTGAAA AAGGAAGAGT ATGAGTATTC AACATTTCCG TGTCGCCCTT

1401  ATTCCCTTTT TTGCGGCATT TTGCCTTCCT GTTTTTGCTC ACCCAGAAAC GCTGGTGAAA GTAAAAGATG CTGAAGATCA GTTGGGTGCA CGAGTGGGTT

                                                           XmnI
                                                        -----+-----
1501  ACATCGAACT GGATCTCAAC AGCGGTAAGA TCCTTGAGAG TTTTCGCCCC GAAGAACGTT TTCCAATGAT GAGCACTTTT AAAGTTCTGC TATGTGGCGC

                                                                      ScaI
                                                                     ---+--
1601  GGTATTATCC CGTATTGACG CCGGGCAAGA GCAACTCGGT CGCCGCATAC ACTATTCTCA GAATGACTTG GTTGAGTACT CACCAGTCAC AGAAAAGCAT

1701  CTTACGGATG GCATGACAGT AAGAGAATTA TGCAGTGCTG CCATAACCAT GAGTGATAAC ACTGCGGCCA ACTTACTTCT GACAACGATC GGAGGACCGA

1801  AGGAGCTAAC CGCTTTTTTG CACAACATGG GGGATCATGT AACTCGCCTT GATCGTTGGG AACCGGAGCT GAATGAAGCC ATACCAAACG ACGAGCGTGA

1901  CACCACGATG CCTGTAGCAA TGGCAACAAC GTTGCGCAAA CTATTAACTG GCGAACTACT TACTCTAGCT TCCCGGCAAC AATTAATAGA CTGGATGGAG
```

```
                                              BglI                                                   BsaI
                                          -------+----                                             -------
2001  GCGGATAAAG TTGCAGGACC ACTTCTGCGC TCGGCCCTTC CGGCTGGCTG GTTTATTGCT GATAAATCTG GAGCCGGTGA GCGTGGGTCT CGCGGTATCA

2101  TTGCAGCACT GGGGCCAGAT GGTAAGCCCT CCCGTATCGT AGTTATCTAC ACGACGGGGA GTCAGGCAAC TATGGATGAA CGAAATAGAC AGATCGCTGA

2201  GATAGGTGCC TCACTGATTA AGCATTGGTA ACTGTCAGAC CAAGTTTACT CATATATACT TTAGATTGAT TTAAAACTTC ATTTTTAATT TAAAAGGATC

2301  TAGGTGAAGA TCCTTTTTGA TAATCTCATG ACCAAAATCC CTTAACGTGA GTTTTCGTTC CACTGAGCGT CAGACCCCGT AGAAAAGATC AAAGGATCTT

2401  CTTGAGATCC TTTTTTTCTG CGCGTAATCT GCTGCTTGCA AACAAAAAAA CCACCGCTAC CAGCGGTGGT TTGTTTGCCG GATCAAGAGC TACCAACTCT

2501  TTTTCCGAAG GTAACTGGCT TCAGCAGAGC GCAGATACCA AATACTGTTC TTCTAGTGTA GCCGTAGTTA GGCCACCACT TCAAGAACTC TGTAGCACCG

                                          AlwNI
                                        ------+--
2601  CCTACATACC TCGCTCTGCT AATCCTGTTA CCAGTGGCTG CTGCCAGTGG CGATAAGTCG TGTCTTACCG GGTTGGACTC AAGACGATAG TTACCGGATA

2701  AGGCGCAGCG GTCGGGCTGA ACGGGGGGTT CGTGCACACA GCCCAGCTTG GAGCGAACGA CCTACACCGA ACTGAGATAC CTACAGCGTG AGCTATGAGA

2801  AAGCGCCACG CTTCCCGAAG GGAGAAAGGC GGACAGGTAT CCGGTAAGCG GCAGGGTCGG AACAGGAGAG CGCACGAGGG AGCTTCCAGG GGGAAACGCC

2901  TGGTATCTTT ATAGTCCTGT CGGGTTTCGC CACCTCTGAC TTGAGCGTCG ATTTTTGTGA TGCTCGTCAG GGGGGCGGAG CCTATGGAAA AACGCCAGCA

3001  ACGCGGCCTT TTTACGGTTC CTGGCCTTTT GCTGGCCTTT TGCTCACATA GTCATGCCCC GCGCCCACCG GAAGGAGCTG ACTGGGTTGA AGGCTCTCAA

3101  GGGCATCGGT CGAGATCCCG GTGCCTAATG AGTGAGCTAA CTTACATTAA TTGCGTTGCG CTCACTGCCC GCTTTCCAGT CGGGAAACCT GTCGTGCCAG

                                                             BbeI
                                                           ------+
                                                             SfoI
                                                           ----+--
                                                             NarI
                                                           ---+---
                                                             KasI
                                                           --+----
3201  CTGCATTAAT GAATCGGCCA ACGCGCGGGG AGAGGCGGTT TGCGTATTGG GCGCCAGGGT GGTTTTTCTT TTCACCAGTG AGACGGGCAA CAGCTGATTG

                                                                                                   HpaI
                                                                                                  ---+--
3301  CCCTTCACCG CCTGGCCCTG AGAGAGTTGC AGCAAGCGGT CCACGCTGGT TTGCCCCAGC AGGCGAAAAT CCTGTTTGAT GGTGGTTAAC GGCGGGATAT

                                                   EcoRV                                   BssHII
                                                  ---+--                                  -+-----
3401  AACATGAGCT GTCTTCGGTA TCGTCGTATC CCACTACCGA GATATCCGCA CCAACGCGCA GCCCGGACTC GGTAATGGCG CGCATTGCGC CCAGCGCCAT

3501  CTGATCGTTG GCAACCAGCA TCGCAGTGGG AACGATGCCC TCATTCAGCA TTTGCATGGT TTGTTGAAAA CCGGACATGG CACTCCAGTC GCCTTCCCGT

                                                                                                ApaI
                                                                                               -----+
                                                                                               PspOMI
                                                                                               -+----
3601  TCCGCTATCG GCTGAATTTG ATTGCGAGTG AGATATTTAT GCCAGCCAGC CAGACGCAGA CGCGCCGAGA CAGAACTTAA TGGGCCCGCT AACAGCGCGA

             BstEII
            -+------
3701  TTTGCTGGTG ACCCAATGCG ACCAGATGCT CCACGCCCAG TCGCGTACCG TCTTCATGGG AGAAAATAAT ACTGTTGATG GGTGTCTGGT CAGAGACATC

                                                                                                       AflIII
                                                                                                      -+-----
                                                                                        BclI            MluI
                                                                                       -+----         -+-----
3801  AAGAAATAAC GCCGGAACAT TAGTGCAGGC AGCTTCCACA GCAATGGCAT CCTGGTCATC CAGCGGATAG TTAATGATCA GCCCACTGAC GCGTTGCGCG

3901  AGAAGATTGT GCACCGCCGC TTTACAGGCT TCGACGCCGC TTCGTTCTAC CATCGACACC ACCACGCTGG CACCCAGTTG ATCGGCGCGA GATTTAATCG

4001  CCGCGACAAT TTGCGACGGC GCGTGCAGGG CCAGACTGGA GGTGGCAACG CCAATCAGCA ACGACTGTTT GCCCGCCAGT TGTTGTGCCA CGCGGTTGGG

4101  AATGTAATTC AGCTCCGCCA TCGCCGCTTC CACTTTTTCC CGCGTTTTCG CAGAAACGTG GCTGGCCTGG TTCACCACGC GGGAAACGGT CTGATAAGAG

               BstAPI
            --------+---
4201  ACACCGGCAT ACTCTGCGAC ATCGTATAAC GTTACTGGTT TCACATTCAC CACCCTGAAT TGACTCTCTT CCGGGCGCTA TCATGCCATA CCGCGAAAGG

                                                                   EcoNI
                                                               -----+------
4301  TTTTGCGCCA TTCGATGGTG TCCGGGATCT CGACGCTCTC CCTTATGCGA CTCCTGCATT AGGAAGCAGC CCAGTAGTAG GTTGAGGCCG TTGAGCACCG

4401  CCGCCGC
```

(B)

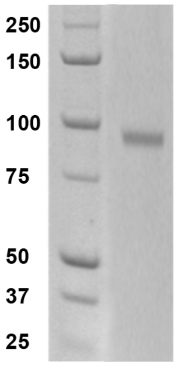

Fig. S2. (A) A fully annotated plasmid map of the pColdI vector system used to sub–clone the final DNA sequence for expression in *E. coli*. (B) A representative SDS–PAGE gel of the HIHA-Scl2 protein.

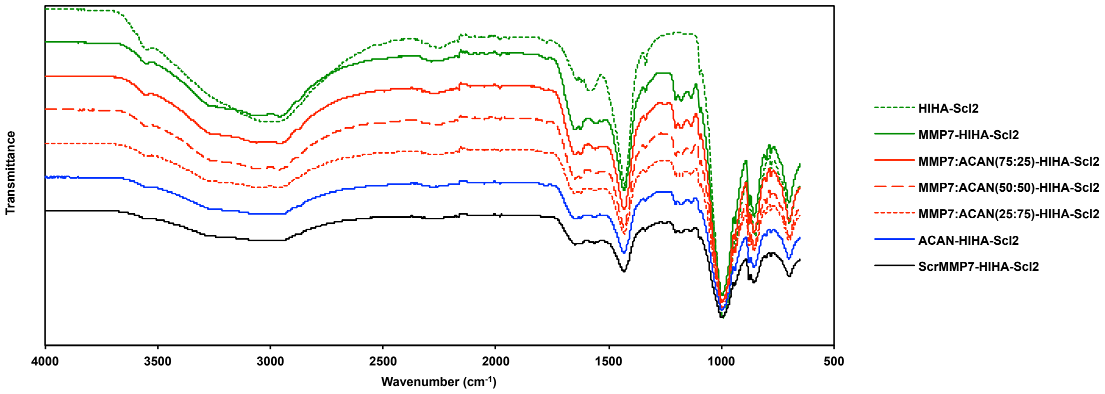

Fig. S3. Representative FTIR spectra of functionalized Scl2 proteins confirming the conjugation of Scl2 with acrylate–functionalized MMP7 and ACAN peptides.

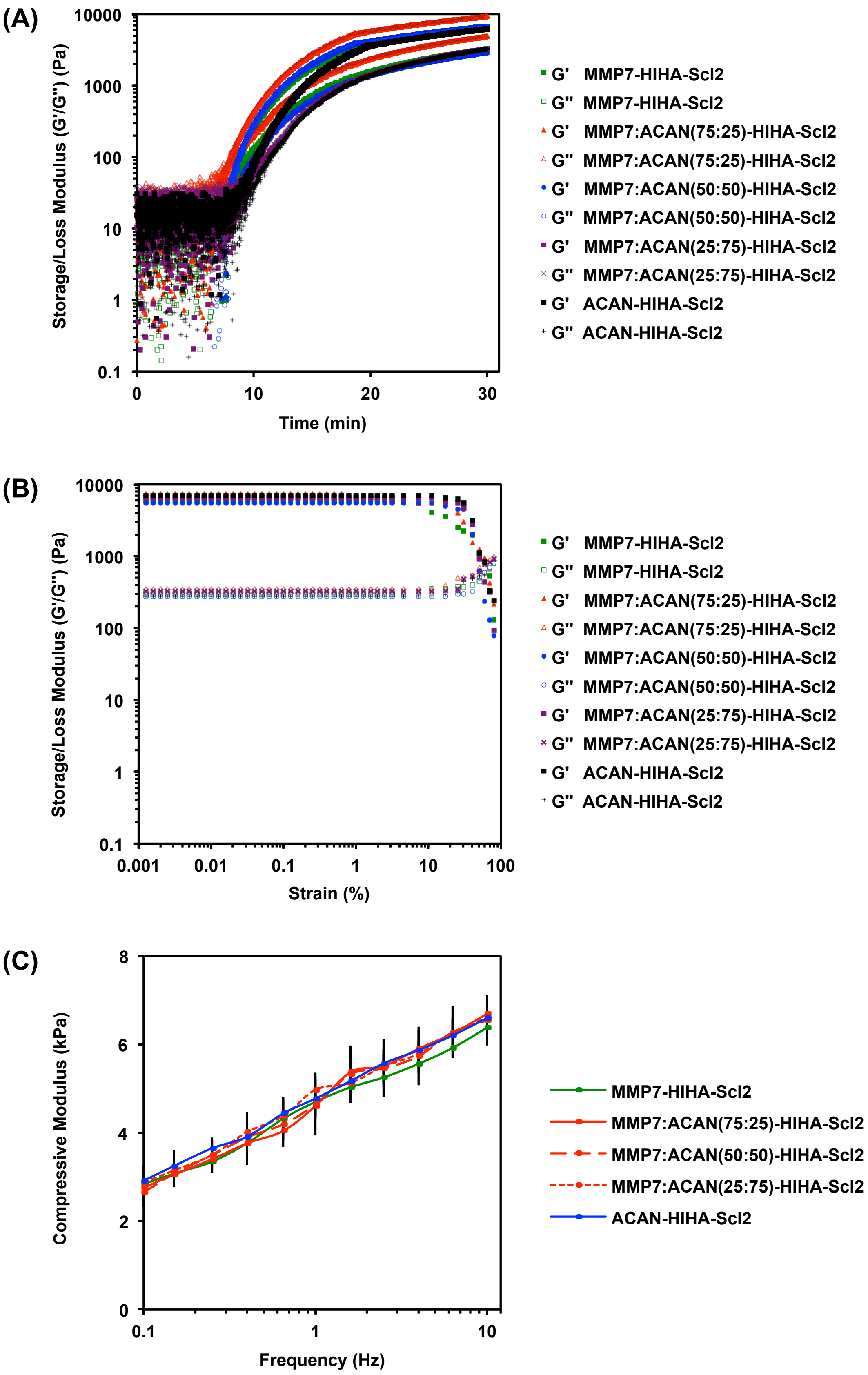

Fig. S4. Rheological properties of acellular functionalized Scl2 hydrogels. (A) Time to gelation determined at a temperature of 37 °C, angular frequency of 6.28 rad/s, and strain of 0.5% shown as G' and G''. (B) Strain sweep at a temperature of 37 °C and an angular frequency of 6.28 rad/s shown as G' and G''. (C) Dynamic mechanical analysis (DMA) used to determine the elastic modulus in unconfined compression of hydrogels compressed to 10% strain at 0.5% strain/min from 0.1 to 10 Hz. Values represent means ± SD (n = 3).

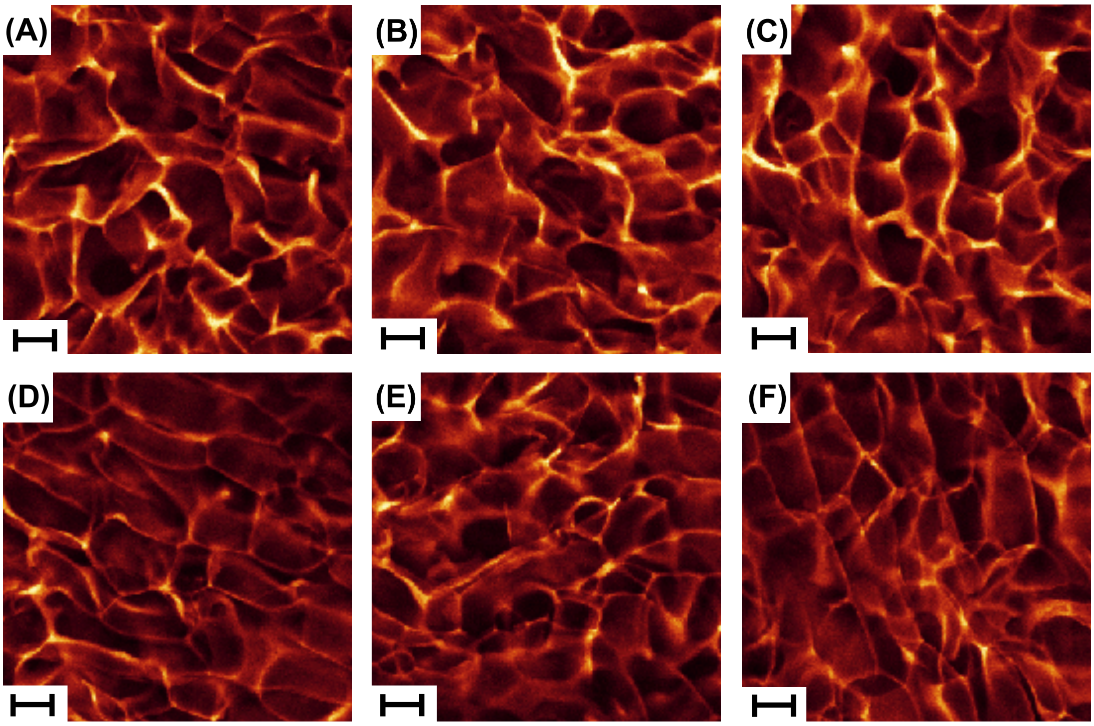

Fig. S5. Representative multi–photon second harmonic generation (MP–SHG) images of acellular (A) ScrMMP7-HIHA-Scl2, (B) MMP7-HIHA-Scl2, (C) MMP7:ACAN(75:25)-HIHA-Scl2, (D) MMP7:ACAN(50:50)-HIHA-Scl2, (E) MMP7:ACAN(25:75)-HIHA-Scl2, and (F) ACAN-HIHA-Scl2 hydrogels (scale bars are 10 μm).

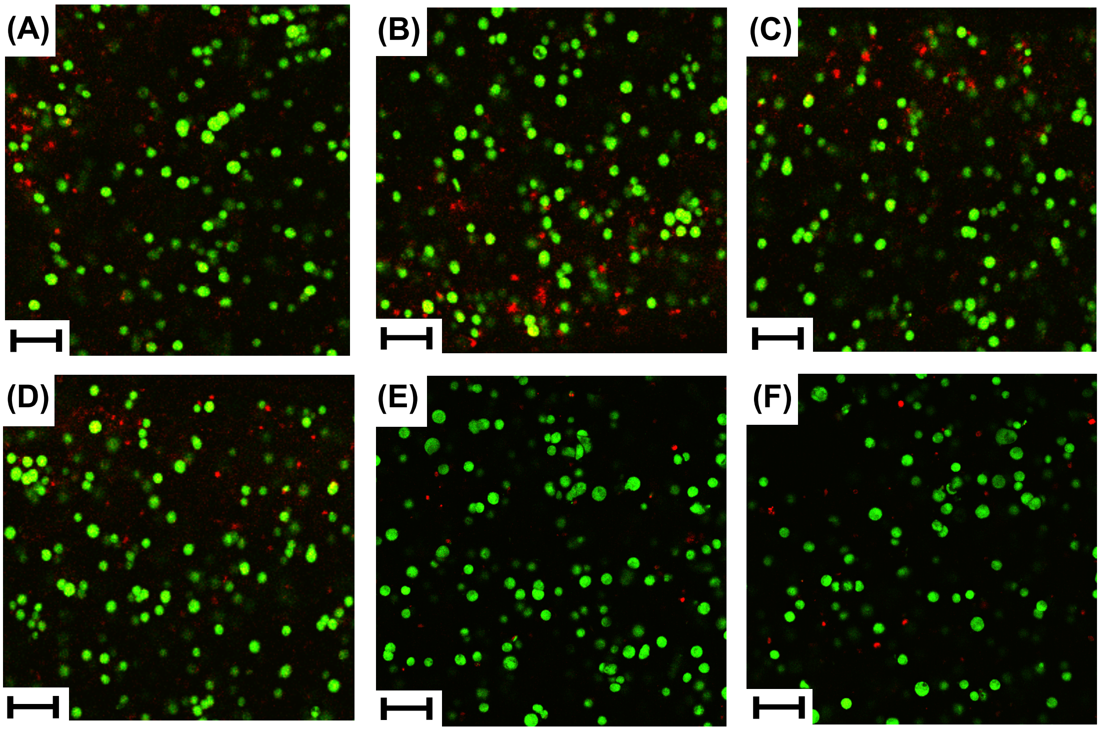

Fig. S6. hMSC viability in Scl2 hydrogels. LIVE/DEAD® Viability/Cytotoxicity assay on hMSCs cultured for 6 weeks in hydrogels. Representative confocal images of cells in (A) ScrMMP7-HIHA-Scl2, (B) MMP7-HIHA-Scl2, (C) MMP7:ACAN(75:25)-HIHA-Scl2, (D) MMP7:ACAN(50:50)-HIHA-Scl2, (E) MMP7:ACAN(25:75)-HIHA-Scl2, and (F) ACAN-HIHA-Scl2 hydrogels (scale bars are 50 μm).

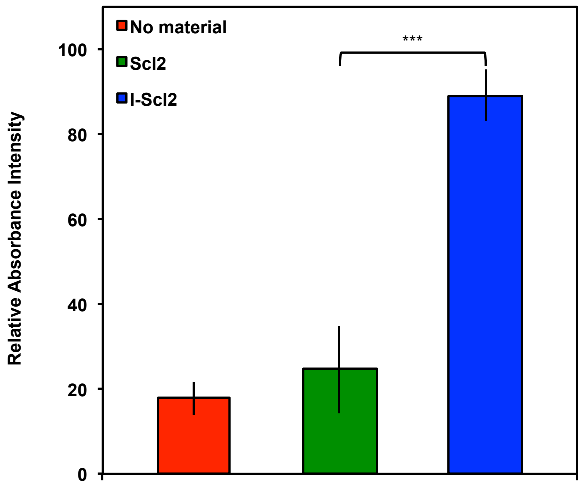

Fig. S7. Integrin (α1β1 and α2β1) binding of hMSCs on Scl2 proteins. Empty wells were used as a negative control denoted 'no material'. Values represent means ± SD. ***$p < 0.001$ (n = 3 for each donor; 3 different bone marrow–derived hMSC donors).

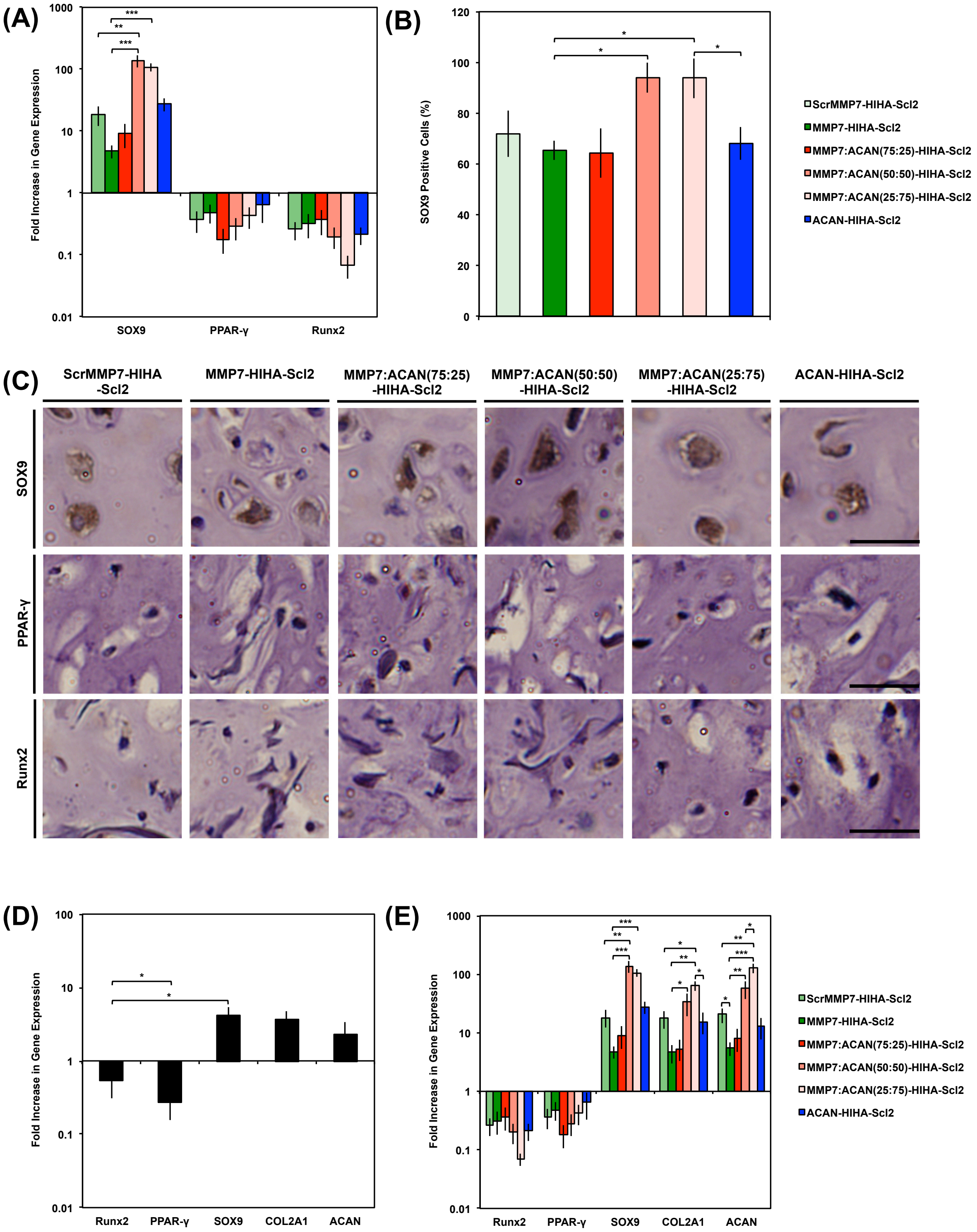

Fig. S8. (A) hMSC gene expression in Scl2 hydrogels. SOX9, Runx2, and PPAR-γ gene expression of hMSCs encapsulated in hydrogels after 2 weeks in culture, as analyzed using the ΔΔCt method. Data presented as a fold difference relative to undifferentiated hMSCs (calibrator) prior to encapsulation and normalized to GAPDH (housekeeping gene). (B) Percentage positive SOX9 cells determined from immunohistochemical stained sections for SOX9 and normalized to total number of cells. (C) High magnification images showing representative immunohistochemical examination of hMSC–seeded hydrogels after 14 days in culture. Hydrogels are stained for SOX9, PPAR-γ, and Runx2, respectively, from top to bottom. Scale bars are 100 µm. (D) hMSC gene expression in pellet cultures. Runx2, PPAR-γ, SOX9, COL2A1, and ACAN gene expression of hMSCs after 2 weeks in culture, as analyzed using the ΔΔCt method. Data presented as a fold difference relative to undifferentiated hMSCs (calibrator) and normalized to GAPDH (housekeeping gene). (E) hMSC gene expression in Scl2 hydrogels. Runx2, PPAR-γ, SOX9, COL2A1, and ACAN gene expression of hMSCs encapsulated in hydrogels after 2 weeks in culture, as analyzed using the ΔΔCt method. Data presented as a fold difference relative to undifferentiated hMSCs (calibrator) prior to encapsulation and normalized to GAPDH (housekeeping gene). The data in Fig. S8 is reproduced from Fig. 6. Values represent means ± SD. $^{**}p < 0.01$, $^{***}p < 0.001$ (n = 3 for each donor; 3 different bone marrow–derived hMSC donors).

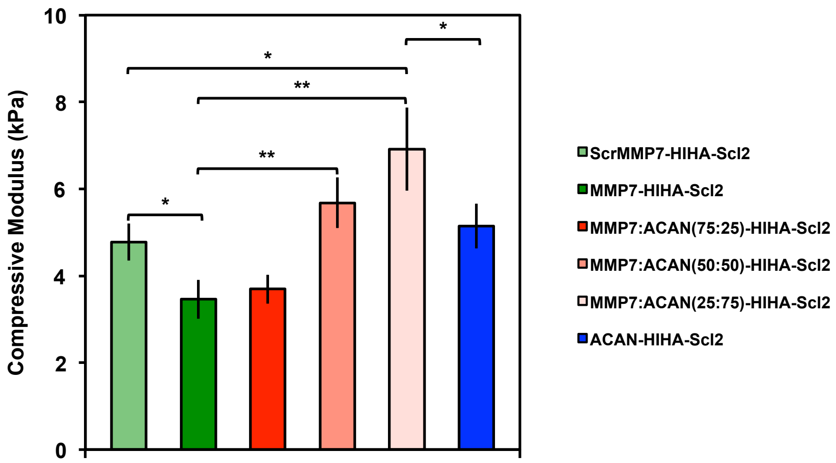

Fig. S9. Dynamic mechanical analysis (DMA). Elastic compression moduli of cell–seeded Scl2 hydrogels compressed to 10% strain at 0.5% strain/min and 1 Hz after 6 weeks of culture. Values represent means ± SD. (n = 3 for each donor; 3 different bone marrow–derived hMSC donors).

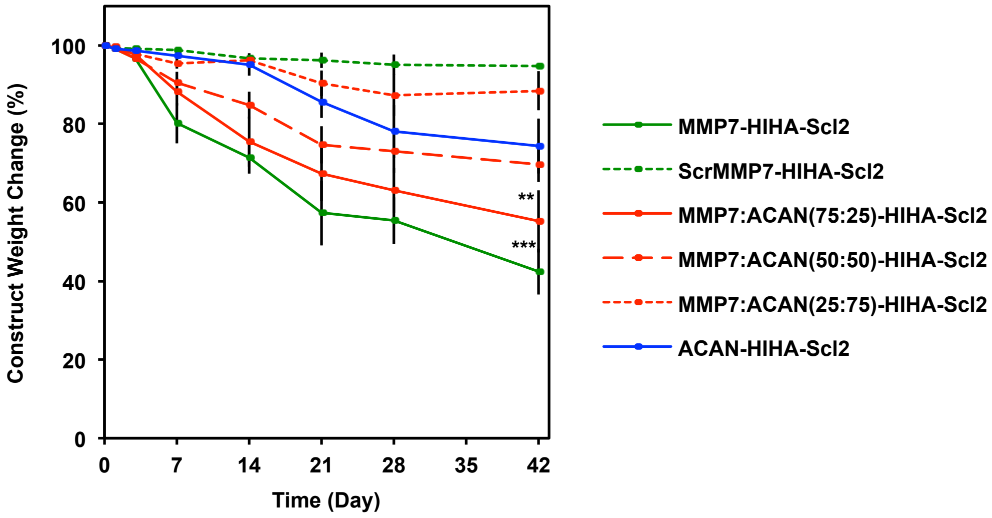

Fig. S10. Scl2 hydrogel dry weight change over time in culture with hMSCs. Weight change was normalized to dry weight at day 0. Values represent means ± SD. $^{**}p < 0.01$ versus MMP7:ACAN(25:75)-HIHA-Scl2, $^{***}p < 0.001$ versus MMP7:ACAN(25:75)-HIHA-Scl2 (n = 3 for each donor; 3 different bone marrow–derived hMSC donors).

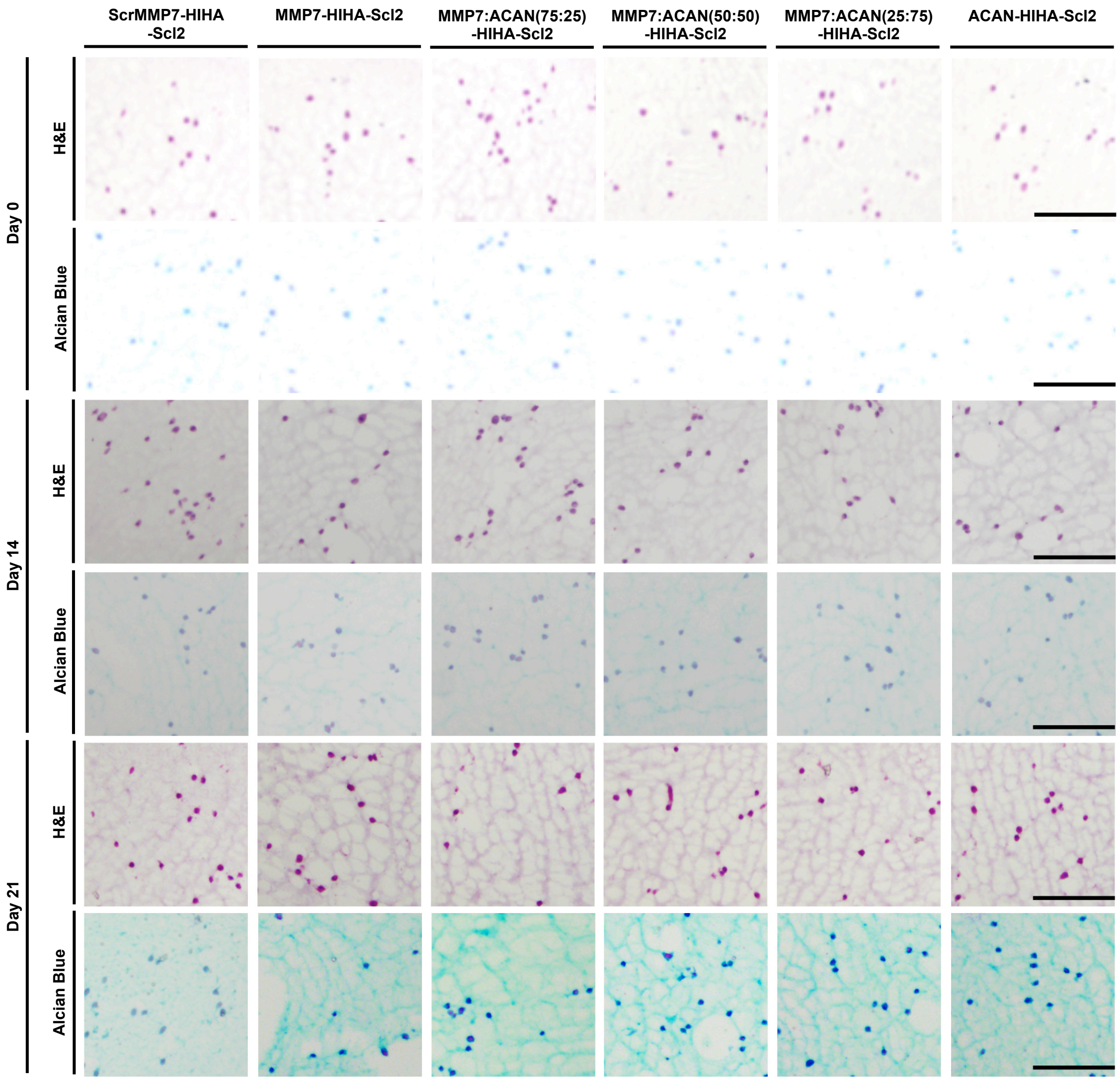

Fig. S11. ECM accumulation of hMSC–seeded Scl2 hydrogels. Representative histological examination of hMSC–seeded hydrogels after 0, 14, and 21 days in culture. Hydrogels are stained with haematoxylin and eosin (H&E) and alcian blue for sGAG. Scale bars are 50 μm.

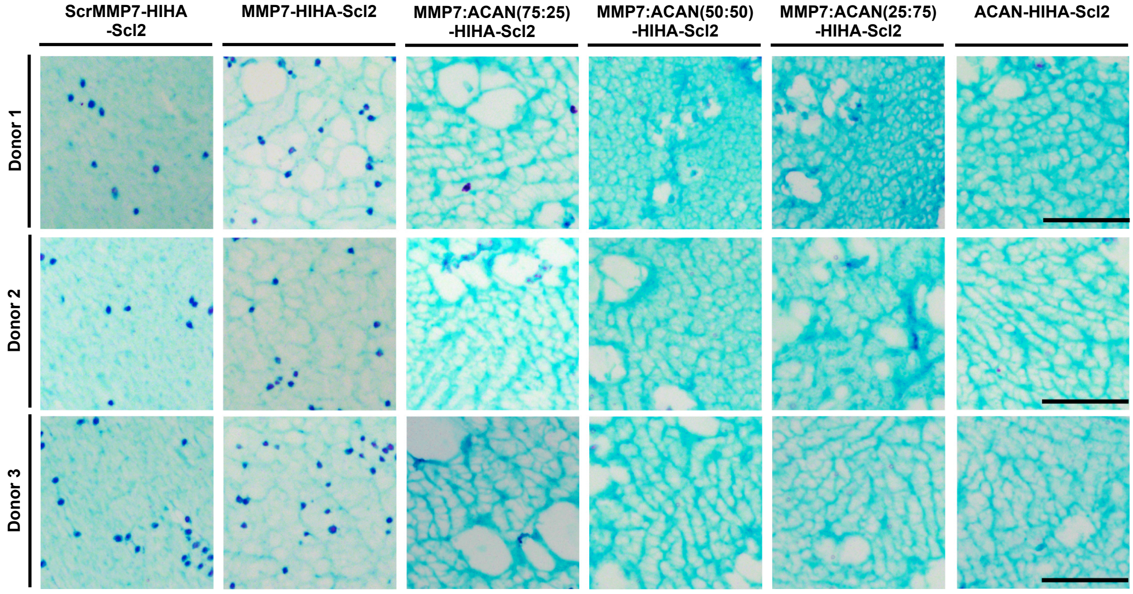

Fig. S12. ECM accumulation of hMSC–seeded Scl2 hydrogels from three different donors. Representative histological examination of hMSC–seeded hydrogels after 42 days in culture. Donor 1 images are reproduced from Fig. 7. Hydrogels are stained with alcian blue for sGAG. Scale bars are 50 μm.

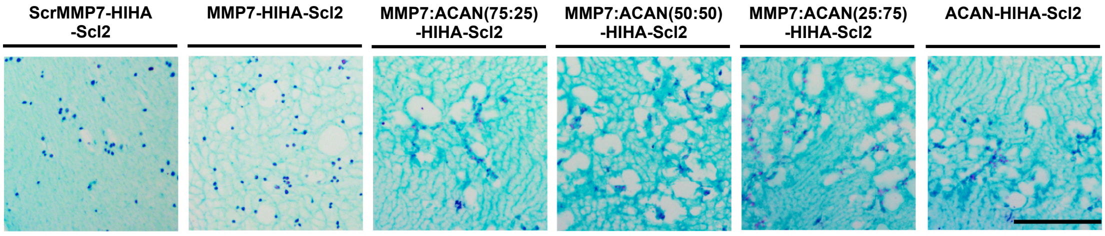

Fig. S13. Low magnification images demonstrating ECM accumulation of hMSC–seeded Scl2 hydrogels. Representative histological examination of hMSC–seeded hydrogels after 6 weeks in culture. Hydrogels are stained with alcian blue for sGAG. Scale bars are 200 µm.

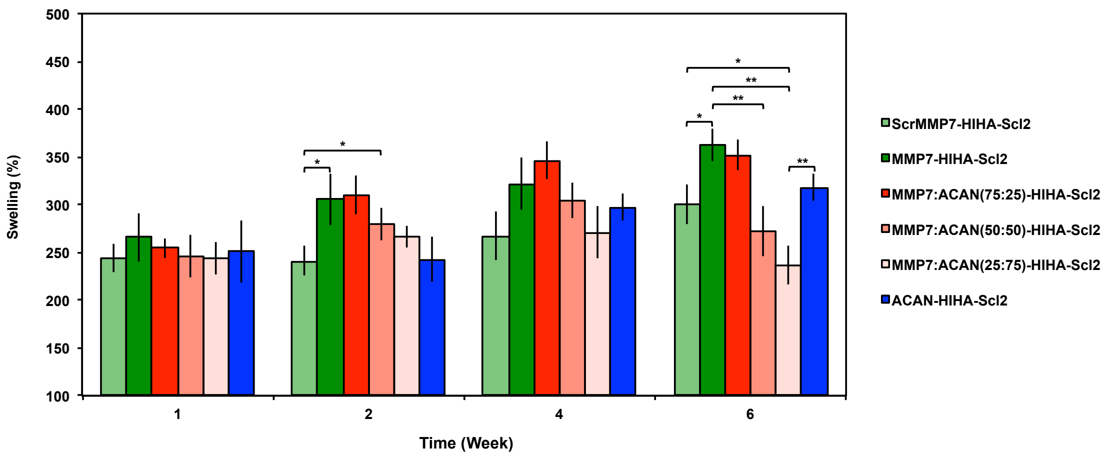

Fig. S14. Swelling behavior of cell–seeded Scl2 hydrogels over time. Values represent means ± SD. *$p < 0.05$, **$p < 0.01$ (n = 3 for each donor; 3 different bone marrow–derived hMSC donors).
